# Revealing the Complexity of Sweepovirus-Deltasatellite–Plant Host Interactions: Expanded Natural and Experimental Helper Virus Range and Effect Dependence on Virus-Host Combination

**DOI:** 10.3390/microorganisms9051018

**Published:** 2021-05-10

**Authors:** Camila G. Ferro, F. Murilo Zerbini, Jesús Navas-Castillo, Elvira Fiallo-Olivé

**Affiliations:** 1 Instituto de Hortofruticultura Subtropical y Mediterránea “La Mayora”, Consejo Superior de Investigaciones Científicas–Universidad de Málaga (IHSM-CSIC-UMA), 29750 Algarrobo-Costa, Málaga, Spain; cgfufv@hotmail.com; 2 Departmento de Fitopatologia/BIOAGRO, Universidade Federal de Viçosa, Viçosa 36570-900, MG, Brazil; zerbini@ufv.br; 3 National Research Institute for Plant-Pest Interactions, Universidade Federal de Viçosa, Viçosa 36570-900, MG, Brazil

**Keywords:** *Geminiviridae*, *Begomovirus*, sweepoviruses, DNA satellites, *Deltasatellite*, helper virus range, transreplication

## Abstract

Sweepoviruses are begomoviruses (genus *Begomovirus*, family *Geminiviridae*) with ssDNA genomes infecting sweet potato and other species of the family Convolvulaceae. Deltasatellites (genus *Deltasatellite*, family *Tolecusatellitidae*) are small-size non-coding DNA satellites associated with begomoviruses. In this study, the genetic diversity of deltasatellites associated with sweepoviruses infecting *Ipomoea indica* plants was analyzed by further sampling the populations where the deltasatellite sweet potato leaf curl deltasatellite 1 (SPLCD1) was initially found, expanding the search to other geographical areas in southern continental Spain and the Canary Islands. The sweepoviruses present in the samples coinfected with deltasatellites were also fully characterized by sequencing in order to define the range of viruses that could act as helper viruses in nature. Additionally, experiments were performed to assess the ability of a number of geminivirids (the monopartite tomato leaf deformation virus and the bipartite NW begomovirus Sida golden yellow vein virus, the bipartite OW begomovirus tomato leaf curl New Delhi virus, and the curtovirus beet curly top virus) to transreplicate SPLCD1 in their natural plant hosts or the experimental host *Nicotiana benthamiana*. The results show that SPLCD1 can be transreplicated by all the geminivirids assayed in *N. benthamiana* and by tomato leaf curl New Delhi virus in zucchini. The presence of SPLCD1 did not affect the symptomatology caused by the helper viruses, and its effect on viral DNA accumulation depended on the helper virus–host plant combination.

## 1. Introduction

Begomoviruses (genus *Begomovirus*, family *Geminiviridae*) have circular, single-stranded DNA (ssDNA) genomes composed of one or two genomic components. They are encapsidated in twinned quasi-icosahedral (geminate) particles [1]. Begomoviruses are responsible for many economically important crop diseases worldwide and are transmitted in nature by whiteflies (Hemiptera: Aleyrodidae) of the *Bemisia tabaci* complex [2,3]. The sweepoviruses are begomoviruses infecting sweet potato (*Ipomoea batatas*) and other species of the family Convolvulaceae that group in a cluster basal to the main phylogenetic groups in the genus, the Old World (OW) and the New World (NW) begomoviruses [4,5]. In the last twenty years, a number of sweepoviruses have been identified in various parts of the world, e.g., [5,6,7,8,9,10,11,12,13,14,15]. The sweepoviruses have the typical genomic organization of the monopartite begomoviruses originating from the OW [1,16]. The virion-sense strand encodes the coat protein (CP) with function in particle formation and is essential for viral transmission by *B. tabaci* and the V2 protein that is involved in viral movement. The complementary-sense strand encodes the replication-associated protein (Rep), the replication enhancer protein (REn), the transcriptional activator protein (TrAP), and the C4 protein with diverse functions including virus movement and symptom development. In addition to their specific functions, V2, Rep, TrAP, and C4 proteins have been shown to suppress gene silencing. An intergenic region (IR) has a predicted stem-loop structure and contains the nonanucleotide TAATATTAC conserved among geminivirids and iterons, which are repeated short-sequence motifs close to the TATA box of the Rep promoter that are Rep binding sites and, together with the stem-loop structure, form the origin of virion-sense DNA replication.

Three classes of DNA satellites associated with begomoviruses have been identified: betasatellites [17], alphasatellites [18], and deltasatellites [19]. Deltasatellites contain several genome features: small genome size (about a quarter begomovirus DNA component), lack of coding capacity, two stem-loop structures (one containing a conserved nonanucleotide TAATATTAC and another situated close to begomovirus iteron-like sequences), a short region with high sequence identity with the betasatellite conserver region, and an A-rich region [20]. Deltasatellites are classified in the genus *Deltasatellite* (family *Tolecusatellitidae*), which includes twelve accepted species [21,22,23], three of them include members associated with the sweepovirus sweet potato leaf curl virus (SPLCV) [19,24]. To date, the complex sweepovirus-deltasatellite has been found in the OW (continental Spain, the Spanish Canary Islands, and Portugal) [19,25] and the NW (Venezuela and Puerto Rico) [19,24]. Sweet potato leaf curl deltasatellite 1 (SPLCD1), the first sweepovirus-associated deltasatellite characterized, was found infecting sweet potato in the Canary island of Lanzarote (Spain) and a few blue morning glory (*Ipomoea indica*) plants that were analyzed in a small area of southern continental Spain (Málaga province) [19]. *I. indica* is widely grown ornamentally in the Mediterranean basin, including the coastal areas of Spain, where it is frequently naturalized. Considering the vegetative mode of propagation of sweet potato and *I. indica* plants, the report of SPLCV infecting sweet potato and other *Ipomoea* spp. in several countries around the world, and the fact that at least one of the sweepovirus-associated deltasatellites, SPLCD1, is transmitted by *B. tabaci* [26], the actual distribution of these deltasatellites could be wider than reported.

Available data about diversity and helper virus range of deltasatellites associated with sweepoviruses, and deltasatellites in general, are very limited. Experimentally, it has been shown that SPLCD1 can be transreplicated by two monopartite OW begomoviruses: tomato yellow leaf curl virus (TYLCV) and tomato yellow leaf curl Sardinia virus (TYLCSV) [26]. In some cases, the presence of SPLCD1 reduces the accumulation of the helper begomovirus and symptomatology [26].

In this study, the genetic diversity of deltasatellites associated with sweepoviruses infecting *I. indica* plants was analyzed by further sampling the populations where SPLCD1 was initially found, expanding the search to other geographical areas in southern continental Spain and the Canary Islands of Tenerife and Gran Canaria. The sweepoviruses present in the samples coinfected with deltasatellites were also fully characterized by sequencing in order to define the range of sweepoviruses that could act as helper viruses in nature. Additionally, experiments were performed to assess the ability of a number of geminivirids (a monopartite and a bipartite NW begomovirus, a bipartite OW begomovirus, and a curtovirus) to transreplicate SPLCD1 in their natural plant hosts or the experimental host *Nicotiana benthamiana*. The results show that SPLCD1 was transreplicated by all the geminivirids assayed at least in *N. benthamiana*, that SPLCD1did not affect the symptomatology caused by the helper viruses, and that their effect on viral DNA accumulation depended on the helper virus–host plant combination.

## 2. Materials and Methods

### 2.1. Plant Samples

**Figure 1:**
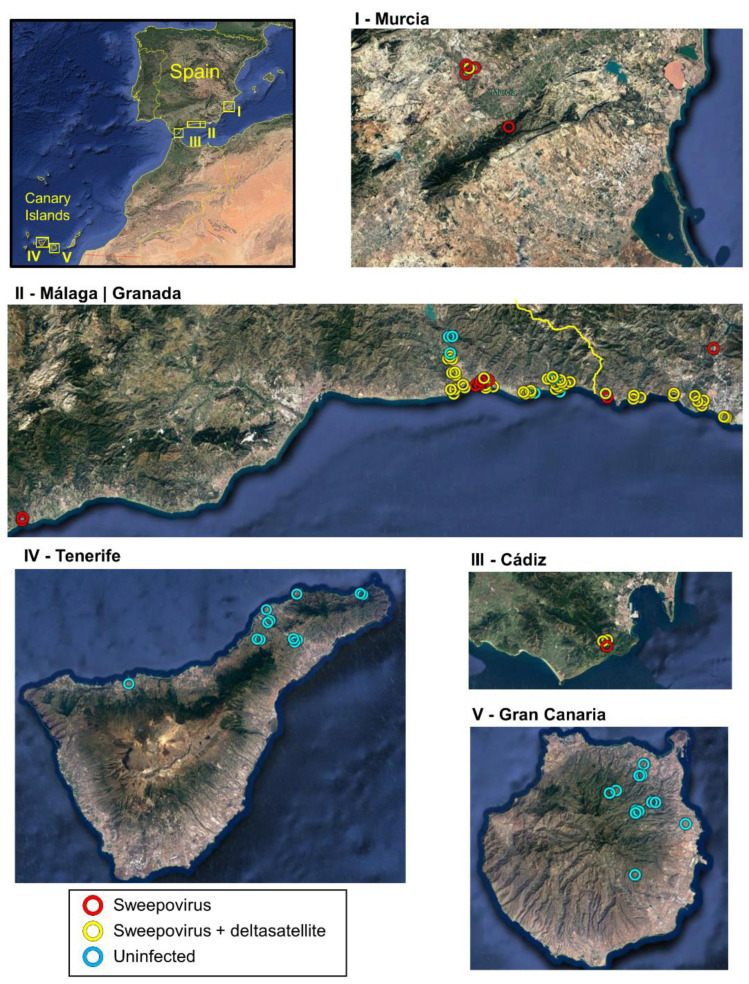
Maps showing the locations of the *Ipomoea indica* plants sampled in southern continental Spain (Murcia (**I**), Granada (**II**), Málaga (**II**), and Cádiz (**III**) provinces) and the Canary Islands (Tenerife (**IV**) and Gran Canaria (**V**)) and analyzed in this work. Samples infected with sweepoviruses (red circles), sweepoviruses plus deltasatellites (yellow circles), and uninfected (blue circles) are indicated in the images.

Leaf samples from 89 *I. indica* plants were collected in southern continental Spain (Murcia, Granada, Málaga, and Cádiz provinces) and the Spanish Canary Islands (Tenerife and Gran Canaria) in 2015 (Figure 1 and Table 1). Each sample consisted of a few leaves that were transported to the laboratory and held at 4 °C until analysis. Samples from the Canary Islands were dried before being transported to the laboratory. Geographical coordinates and presence of leaf symptoms were recorded (Table S1).

### 2.2. DNA Extraction and Cloning

Total DNA was extracted from about 2 cm^2^ leaf tissue using a CTAB-based purification method [27]. Circular ssDNA was amplified by rolling circle amplification (RCA) with ϕ29 DNA polymerase using the TempliPhi DNA Amplification Kit (GE Healthcare, Little Chalfont, UK). Amplified products were initially digested with the restriction enzyme *Hpa*II, a four-base cutter enzyme, to screen for the putative begomovirus-infected samples. Then, RCA products of the selected samples were digested with the six-base cutter restriction enzymes *Bam*HI, *Eco*RI, *Hind*III, *Nco*I, *Pst*I, and *Sac*I to identify those that cleave the begomoviral and deltasatellite genomes at a single site. RCA products of ~2.7 kbp obtained by digestion with *Nco*I, putatively corresponding to begomovirus genomes, were cloned into a covalently closed pGEM-T-Easy Vector (Promega, Madison, WI, USA), while those of ~0.7 kbp digested with *Pst*I, putatively corresponding to deltasatellite genomes, were cloned into pBluescript II SK(+) (Stratagene, San Diego, CA, USA). Inserts of selected clones were sequenced at Macrogen Inc. (Seoul, Korea). 

### 2.3. Sequence Analysis

Sequences were assembled with SeqMan, part of the Lasergene sequence analysis package (DNAStar Inc., Madison, WI, USA) and then analyzed with the BLASTn algorithm [28] for sequence similarity searches in GenBank. Sequences of sweepoviruses and deltasatellites were aligned using MUSCLE [29], and pairwise comparisons of all the sequences obtained in this work and selected sequences retrieved from GenBank (Tables S2 and S3) were carried out with the program Sequence Demarcation Tool (SDT) v. 1.2 [30]. For phylogenetic inference, the maximum likelihood method was used with sequence alignments performed using MUSCLE in MEGA7 [31]. The best-fit model of nucleotide substitution was determined based on corrected Akaike information criterion and Bayesian information criterion as implemented in MEGA7 [31]. The coefficient of evolutionary differentiation of the SPLCV and SPLCD1 genomes obtained in this work and other isolates previously reported from Spain was estimated using the maximum composite likelihood model with the MEGA 7 program [31].

The identification of potential recombinant fragments within sweepovirus and deltasatellites genomes was performed using the seven methods (RDP, GENECONV, BOOTSCAN, MAXIMUM CHI SQUARE, CHIMAERA, SISTER SCAN, and 3SEQ) included in the RDP4 package [32] with default settings from the alignment generated by CLUSTAL V algorithm implemented in MEGA 7 [31]. Only recombination events detected using at least four methods with *p*-values lower than 10^−2^ were considered.

### 2.4. Plant Agroinoculation

For agroinoculation assays, *Agrobacterium tumefaciens* cultures harboring each construct were added at 1:1000 dilution to YEP liquid media containing kanamycin (50 µg/mL) and rifampicin (50 µg/mL) and grown for 2 days at 28 °C. Cultures were centrifuged at 3100 g for 20 min at room temperature and then resuspended in 10 mM MES (pH 5.6), 10 mM MgCl_2_, and 150 µM acetosyringone, adjusting optical density at 600 nm to 1. Infectious clones of SPLCV and SPLCD1 [26], tomato leaf curl New Delhi virus-Spain (ToLCNDV-ES) (a cucurbit-adapted strain hereinafter called simply “tomato leaf curl New Delhi virus” or ”ToLCNDV”) DNA-A and DNA-B [33], Sida golden yellow vein virus (SiGYVV) DNA-A and DNA-B [34], tomato leaf deformation virus (ToLDeV) [35], and beet curly top virus (BCTV) [36] have been described previously. Plants inoculated with *A. tumefaciens* C58C1 cultures containing empty vector (mock) served as negative controls.

*N. benthamiana* at the four-leaf stage and tomato cv. Moneymaker, *Malvastrum coromandelianum*, and zucchini cv. Milenio plants at the two-leaf stage were inoculated with *A. tumefaciens* cultures containing clones of viral DNA components and SPLCD1 by stem puncture inoculation. For that, 0.2 mL of *A. tumefaciens* culture was expelled from a 1 mL syringe fitted with a 27G × 1/2″ needle into three puncture wounds made in the stem. Inoculated plants were maintained in an insect-free growth chamber (25 °C during the day and 18 °C at night, 70% relative humidity, with a 16 h photoperiod at 250 µmoL s^−1^ m^−2^ of photosynthetically active radiation) until analyzed. At least two independent experiments were performed for each virus-deltasatellite combination.

### 2.5. Virus and Deltasatellite Detection and Quantification

For molecular hybridization assays, apical leaves of agroinoculated plants were used for tissue blot of petiole cross-sections (tissue printing) performed on positively charged nylon membranes at 28 days post-inoculation. Hybridization was carried out as previously described [34] using digoxigenin-labelled DNA probes specific to SPLCD1 [26] and each genomic component of SiGYVV [34], ToLDeV [35], ToLCNDV [33], and BCTV [34]. The probes were prepared by PCR according to the DIG-labelling detection kit (Roche Diagnostics, Mannheim, Germany). Plants were visually evaluated periodically for symptoms.

For relative quantitative real-time PCR, total DNA was extracted from the leaves used for tissue printing using the DNeasy Plant Mini Kit (Qiagen, Madison, WI, USA). Several pairs of both forward and reverse PCR primers were designed using the PrimerQuest Tool (Integrated DNA Technology, Coralville, IA, USA) and tested for specificity using a standard curve obtained by serial dilution of known quantities of plasmids containing one copy of each viral genome component or the deltasatellite. Additionally, efficiencies of PCR amplification were tested to be close to 100% to select the primers finally used for the assays (Table S4). Reactions were conducted in a QuantStudio 5 Real-Time PCR System (Applied Biosystems, Foster City, CA, USA). For that, 1 µL of total DNA was analyzed using the PowerUp SYBR Green Master Mix (Thermo Fisher Scientific, Waltham, MA, USA). PCR reactions were performed as follows: 50 °C for 2 min, 95 °C for 2 min, and 40 cycles of 95 °C for 15 s, 56.5 °C for 15 s and 72 °C for 1 min. Each sample was analyzed in triplicate and virus and deltasatellite genomes were quantified by the 2^−ΔΔCt^ method [37], normalizing the amount of target DNA to the amount of plant reference gene DNA (protein phosphatase 2A gene for *N. benthamiana* [38] and elongation factor-1α for zucchini [39]).

Statistical analyses and graphing to compare the effect of the deltasatellite on geminivirid accumulation were performed using Graphpad Prism 6.0 software (GraphPad Software Inc., San Diego, CA, USA). Unpaired *t* test with Welch’s correction or Mann–Whitney test were used, respectively, depending on normal or not normal data distribution (Kolmogorov–Smirnov test). Outlier values were identified by the ROUT method. Differences between means were considered significant when *p* ≤ 0.05.

## 3. Results

### 3.1. Widespread Presence of Sweepoviruses and Associated Deltasatellites Infecting Ipomoea indica in Spain

Fifty-nine out of sixty-five *I. indica* samples collected from four provinces in southern continental Spain were putatively infected by geminivirids based on the detection of a ~2800 bp DNA fragment after digestion of the RCA products with *Nco*I. In addition, 46 out of these 59 samples were also putatively infected by deltasatellites based on the detection of a ~700-bp DNA fragment after digestion of the RCA products with *Pst*I (Table 1). From these samples, 35 full-length sweepovirus genomes (GenBank Accession numbers MW574018-MW574052) and 92 deltasatellite molecules (MW587160-MW587196 and MW587198-MW587252) were obtained (Table S1). The 24 *I. indica* samples collected in the Canary Islands of Tenerife and Gran Canaria tested negative both for sweepoviruses and deltasatellites. 

A number of the *I. indica* plants sampled showed yellow vein (19.1%) and/or leaf curling (18.0%) symptoms (Table 1 and Table S1). No relation was established between these symptoms and infection by sweepoviruses and/or deltasatellites.

Pairwise comparisons using the SDT program showed that the sweepovirus genome sequences obtained in this work could be divided in two major groups (Figure S1). One of the groups that contained 29 sequences with 87.6–99.6% nucleotide identity between them showed the highest identities (94.0–98.9%) with sequences of SPLCV isolates previously described in Spain (EF456741, EF456743, EU839576, EU839578, and FJ151200 [5]). Three subgroups, I to III, can be differentiated in this group, showing 96.5–99.6%, 96.4–99.3%, and 92.8–98.4% identity within them, respectively. Thus, in accordance with the current taxonomic guidelines for the genus *Begomovirus* (an isolate having ≥91% nucleotide identity in full-length genome or DNA-A component to an isolate assigned to a recognized species should be considered to belong to that species) [40], these 29 isolates belong to the species *Sweet potato leaf curl virus*. Similarly, the three subgroups could be considered as different strains (≥94% threshold) within that species. The other group consisted of six sequences (MW574021, MW574031, MW574041, MW574043, MW574045, and MW574050) with 94.2–97.7% identity between them that showed the highest identities (92.8–95.8%) with an isolate of sweet potato mosaic virus (SPMV) from Brazil (FJ969831) [13] or with the FJ151200 SPLCV isolate, but all of them showed a ≥91% identity with both SPMV and SPLCV isolates. The fact that these six isolates have ≥91% identity with isolates previously assigned to two different species, *Sweet potato leaf curl virus* and *Sweet potato mosaic virus*, and following the species demarcation criteria in the genus [40], both species should be merged, the species *Sweet potato mosaic virus* being abolished. Further analysis including all available sweepovirus sequences will determine whether the six abovementioned sequences could be considered to belong to a new strain also containing the isolates previously classified in the species *Sweet potato mosaic virus*.

A recombination analysis performed on the sweepovirus genomes obtained in this work, also including closely related SPLCV isolates previously reported from Spain and a SPMV isolate from Brazil using the seven methods included in the RDP4 package [32], revealed a complex recombination pattern for all the sequences (Figure S2A). Thus, 25 different recombination events were identified and statistically supported by at least three methods, each present in 1–14 sequences, making a total of 66 recombinant fragments in the set of sequences analyzed (Figure S2B). The identified recombinant sequences included five out of the six sweepovirus genomes occupying an intermediate phylogenetic position between SPLCV and SPMV isolates.

Sequencing of the 93 *Pst*I clones confirmed that they corresponded to full-length deltasatellite sequences, with one exception that resulted in being a sweepovirus-deltasatellite chimera (see below). Pairwise comparisons using the SDT program showed that the deltasatellite genome sequences obtained in this work were closely related, showing 89.1–100.0% identity between and 90.8–99.9% within sequences of SPLCD1 isolates previously described from Spain and Portugal [19,25] (Figure S3). In accordance with the proposed <91% species demarcation threshold for the genus *Deltasatellite* [22], these 92 deltasatellite isolates should be classified in the species *Sweet potato leaf curl deltasatellite 1*.

**Figure 2:**
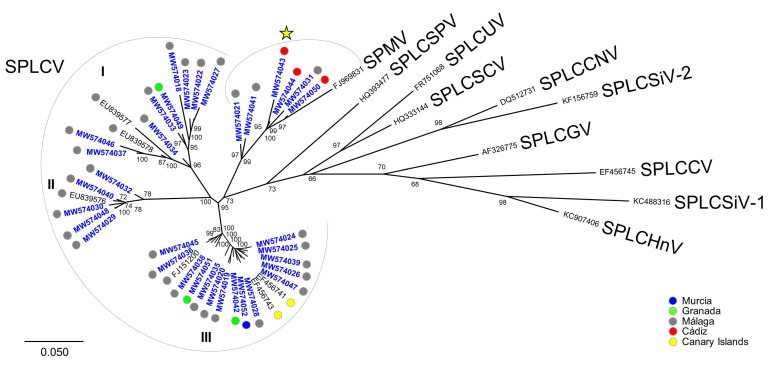
Phylogenetic tree illustrating the relationships of the sweepovirus genomes obtained in this work (highlighted in blue) with closely related sweet potato leaf curl virus (SPLCV) isolates previously reported from Spain and one representative isolate of all other sweepovirus species. Samples were obtained from Murcia (blue dots), Granada (green dots), Málaga (gray dots), and Cádiz (red dots) provinces in southern continental Spain and the Canary Islands (yellow dots). I to III represent major SPLCV clades, and the yellow star corresponds to the clade including sweepovirus described in this work with an intermediate position between SPLCV and sweet potato mosaic virus isolates. The tree was constructed by the maximum likelihood method with the MEGA 7 program using the best fit model, GTR + G+I [31], and bootstrap values (1000 replicates) are shown for supported branches (>70%). The bar below the tree indicates 0.050 nucleotide substitutions per site. Additional details on the sequences and sweepovirus names are included in Tables S1 and S2.

A phylogenetic analysis of all sweepovirus genomes obtained in this work (highlighted in blue in Figure 2) showed them grouped in four clades. Clades I, II, and III also contained SPLCV isolates previously characterized from Spain [5], thus supporting the pairwise sequence identity results described above. A fourth clade (marked with a yellow star in Figure 2) included the six sequences showing a ≥91% identity with both SPLCV and SPMV isolates plus the Brazilian isolate of SPMV (FJ969831) [13]. Thus, the mentioned six sequences somehow occupied an intermediate position between SPLCV and SPMV isolates. This is in agreement with the pairwise comparison results that strongly suggested that the species *Sweet potato mosaic virus* should merge with *Sweet potato leaf curl virus* (Figure S1). No obvious geographical structure was observed for any of the four clades, with isolates from Málaga province present in all of them. Although the low number of sequences from other regions precluded drawing definitive conclusions, the estimate of the coefficient of evolutionary differentiation for each population pair and all populations together showed that in most cases the genetic diversity within populations was higher than among populations (Table S5).

**Figure 3:**
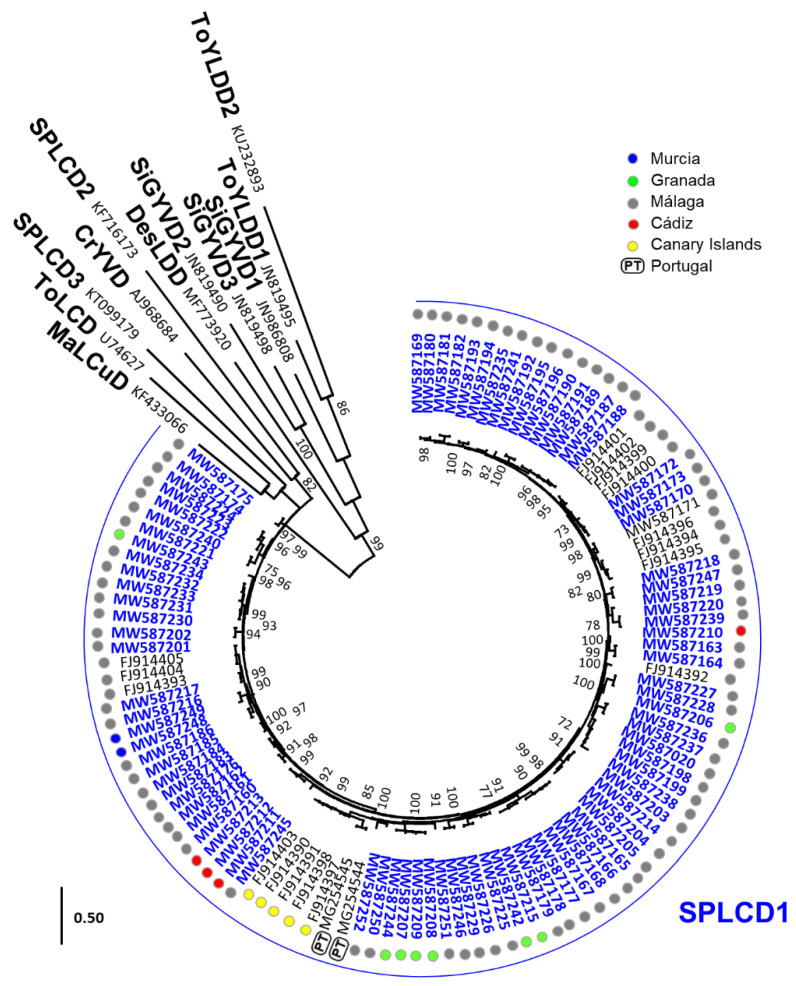
Phylogenetic tree illustrating the relationship of isolates of the deltasatellites obtained in this work (highlighted in blue) with sweet potato leaf curl deltasatellite 1 (SPLCD1) isolates previously reported from Spain and Portugal and one representative isolate of all other deltasatellite species. Samples were obtained from Murcia (blue dots), Granada (green dots), Málaga (gray dots), and Cádiz (red dots) provinces in southern continental Spain and the Canary Islands (yellow dots). The tree was constructed by the maximum likelihood method with the MEGA 7 program using the best fit model T92 + G [31], and bootstrap values (500 replicates) are shown for supported branches (>70%). The bar below the tree indicates 0.50 nucleotide substitutions per site. Additional details on the sequences and deltasatellite names are included in Tables S1 and S3.

A phylogenetic analysis of all deltasatellite sequences available in GenBank including the genomes obtained in this work (highlighted in blue in Figure 3) showed that the SPLCD1 sequences grouped in a single major clade. Numerous minor short-branched clusters, many of them with no bootstrap support, were shown within that major clade. This is in agreement with the pairwise comparison results that showed that the deltasatellites characterized in this work were closely related between them and with the SPLCD1 isolates previously described from Spain and Portugal. As it was shown for SPLCV, no obvious geographical clustering was evident for SPLCD1 isolates, and the estimate of the coefficient of evolutionary differentiation also showed that in most cases the genetic diversity within populations was higher than among populations (Table S5). Moreover, no significant recombination events were identified with RDP4.

### 3.2. Detection of a Sweepovirus-Deltasatellite Chimera

**Figure 4:**
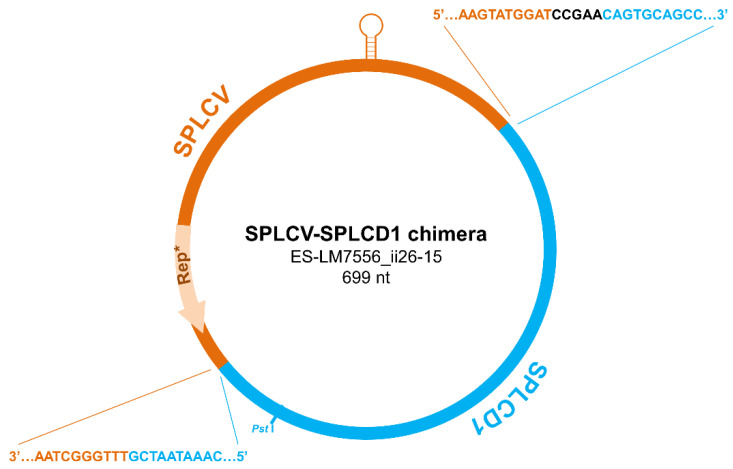
Schematic representation of the chimeric sweepovirus-deltasatellite molecule identified in an *Ipomoea indica* plant from Málaga province (sample ii26). The viral moiety (partial sequence of sweet potato leaf curl virus including a truncated replication-associated protein, Rep *) is represented in orange, and the deltasatellite moiety (partial sequence of sweet potato leaf curl deltasatellite 1) is represented in blue. Nucleotides in black (CCGAA) are not present in the putative parental sequences. The stem-loop structure containing the conserved nanonucleotide TAATATTAC is shown at the top of the graph. The restriction site used for cloning is indicated (*Pst*I).

Sequencing of one of the clones with the characteristic size of deltasatellites resulted to be a sweepovirus-deltasatellite chimera (Figure 4). The chimera was found in sample ii16 collected in Málaga province. The insert of this clone (MW587197) was determined to be 699 bp in length, and a BLASTn search showed significant identity with available sequences of deltasatellites for only about half of the length (coordinates 99–443). This DNA fragment included the right half of the conserved stem-loop structure of the deltasatellite, and the A-rich region and showed 97.4% identity to the equivalent region of a deltasatellite isolated from the same sample (MW587238). BLASTn analysis of the remaining insert (coordinates 448–98) showed significant identity with sweepovirus sequences. This DNA fragment contained the sweepovirus intergenic region (including the stem-loop structure containing the conserved nanonucleotide TAATATTAC) and a truncated replication-associated protein and showed 92.0% identity to the corresponding genome region of the SPLCV isolate cloned from the same sample (MW574045). One of the boundaries between the sweepovirus and the deltasatellite moities of the chimera included four nucleotides not present in the putative parental sequences (CCGAA, in black in Figure 4).

### 3.3. Transreplication of Sweet Potato Leaf Curl Deltasatellite 1 by Old World and New World Begomoviruses as Well as by a Curtovirus in Nicotiana benthamiana Plants

**Figure 5:**
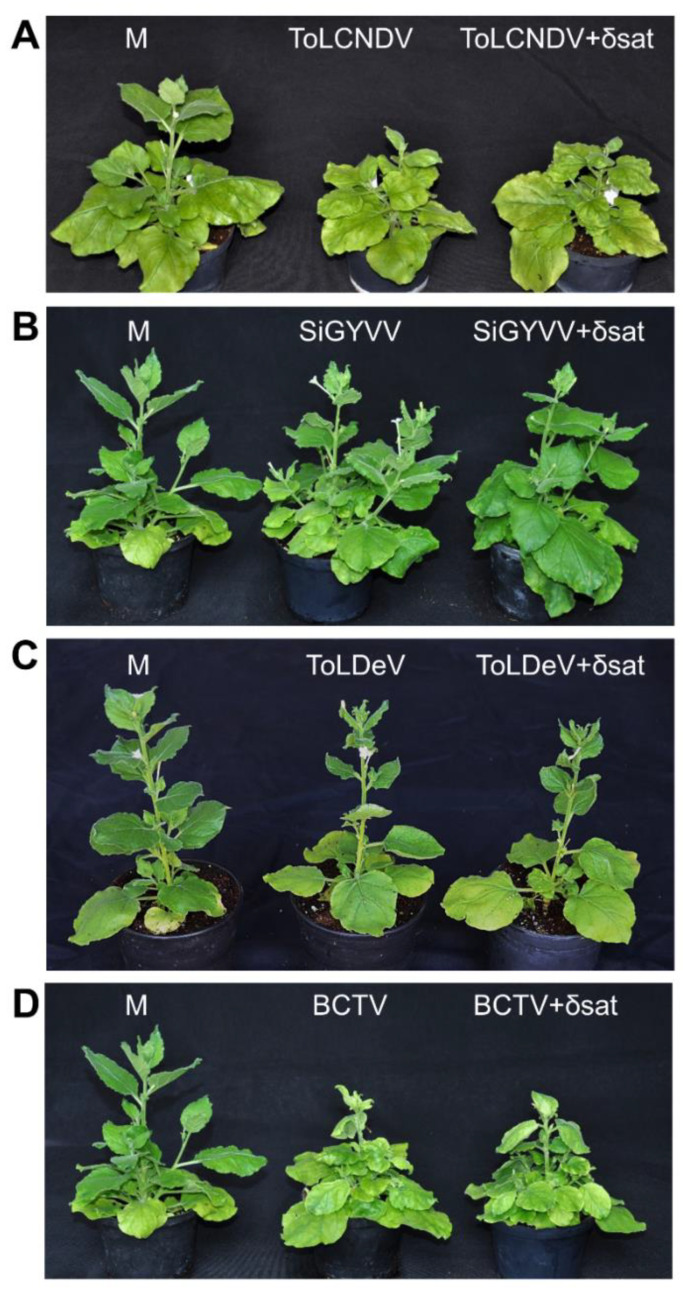
Symptoms caused by (**A**) tomato leaf curl New Delhi virus (ToLCNDV), (**B**) Sida golden yellow vein virus (SiGYVV), (**C**) tomato leaf deformation virus (ToLDeV), and (**D**) beet curly top virus (BCTV) alone or in combination with sweet potato leaf curl deltasatellite 1 (δsat) on agroinoculated *Nicotiana benthamiana* plants. Mock-inoculated plants (M) are shown at the left of each panel. Photographs of representative plants were taken at 30 days post-inoculation.

*N. benthamiana* plants agroinoculated with the geminivirids ToLCNDV (a bipartite OW begomovirus), SiGYVV (a bipartite NW begomovirus), ToLDeV (a monopartite NW begomovirus), or BCTV (a curtovirus) or combinations thereof with SPLCD1 were assessed for virus and deltasatellite accumulation (Table 2, Figure S4) and symptom development (Figure 5).

Agroinoculation with all viruses and virus-deltasatellite combinations resulted in virtually all *N. benthamiana* plants becoming systemically infected by the viruses, as shown by tissue print hybridization of apical leaves with probes specific for each geminivirid genome or for DNA-A and DNA-B components in the case of bipartite begomoviruses (Table 2, Figure S4).

In the case of agroinoculation with ToLCNDV, SiGYVV, or ToLDeV in combination with SPLCD1, all the plants infected by the virus were also infected by the deltasatellite, thus showing that these begomoviruses were able to transreplicate SPLCD1 in *N. benthamiana* plants. In the case of plants inoculated with BCTV plus SPLCD1, the deltasatellite was detected only in 50% of the plants infected with the virus (7 out of 15 in Experiment 1 and 8 out of 15 in Experiment 2), thus showing that the curtovirus BCTV is also able to act as a helper virus for SPLCD1 in this host, although not as efficiently as the begomoviruses tested. Positive (SPLCV, SPLCV + SPLCD1) and negative (mock) control plants became infected and remained healthy, respectively. 

Plants infected by ToLCNDV showed leaf yellowing and mild curling as well as a severe reduction in plant growth (Figure 5A), those by SiGYVV showed mild leaf curling (Figure 5B), those by ToLDeV showed leaf deformation (Figure 5C) and those by BCTV showed leaf yellowing and curling as well as a severe reduction in plant growth (Figure 5D). Co-infection with the deltasatellite did not alter the symptoms caused by each geminivirid.

### 3.4. Transreplication of Sweet Potato Leaf Curl Deltasatellite 1 in the Natural Plant Hosts of Helper Geminivirids

The geminivirids shown to act as helper viruses of SPLCD1 in *N. benthamiana* were also tested for their ability to transreplicate the deltasatellite in some of their natural plant hosts, i.e., zucchini for ToLCNDV, *Malvastrum coromandelianum* for SiGYVV and tomato for ToLDeV and BCTV.

Agroinoculation of tomato plants with ToLDeV or BCTV alone or in combination with SPLCD1 resulted in most of the plants being infected by the virus (Table 3). The plants infected with ToLDeV or BCTV showed leaf deformation and leaf curling, respectively. None of the virus-infected plants became infected by the deltasatellite assessed by tissue print hybridization.

Agroinoculation of *M. coromandelianum* with SiGYVV alone or in combination with SPLCD1 resulted in approximately 50% of the plants being infected by the virus (Table 3), which showed yellow mosaic leaf symptoms. None of the virus-infected plants became infected by the deltasatellite as assessed by tissue print hybridization.

**Figure 6:**
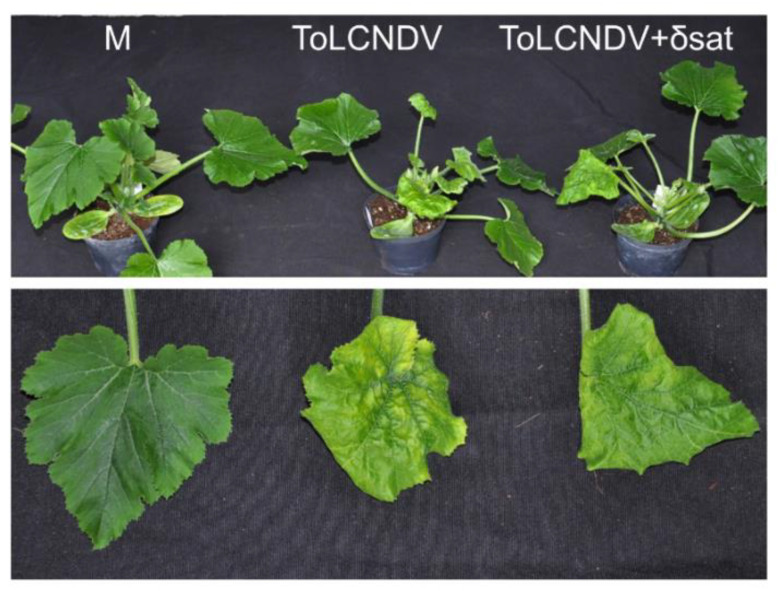
Symptoms caused by tomato leaf curl New Delhi virus (ToLCNDV) alone or in combination with sweet potato leaf curl deltasatellite 1 (δsat) on agroinoculated zucchini plants. Mock-inoculated plants (M) are shown at the left of each panel. Photographs of representative plants were taken at 30 days post-inoculation.

Agroinoculation of zucchini with ToLCNDV alone or in combination with SPLCD1 resulted in almost 100% of the plants being infected by the virus (Table 3, Figure S5), which showed leaf mosaic and curling symptoms (Figure 6). The deltasatellite was detected in approximately 50% of the virus-infected plants (5 out of 15 in Experiment 1 and 9 out of 14 in Experiment 2) (Table 3). The presence of the deltasatellite did not modify the symptoms caused by ToLCNDV (Figure 6).

### 3.5. Effect of Sweet Potato Leaf Curl Deltasatellite 1 on Accumulation of Helper Geminivirids: Dependence on the Virus-Host Combination

**Figure 7:**
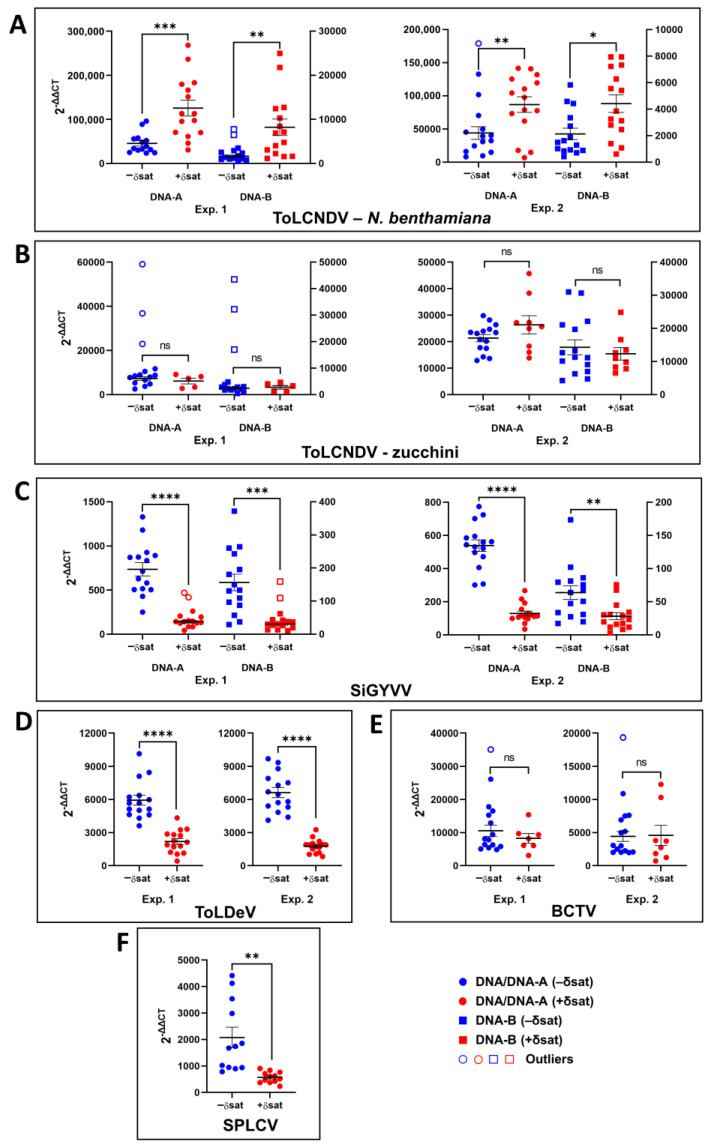
Relative quantification by real-time PCR of helper geminivirids alone (–δsat) or in combination with sweet potato leaf curl deltasatellite 1 (+δsat). (**A**,**B**) Tomato leaf curl New Delhi virus (ToLCNDV) DNA-A and DNA-B in *Nicotiana benthamiana* and zucchini, respectively. (**C**) Sida golden yellow vein virus (SiGYVV) DNA-A and DNA-B, (**D**) tomato leaf deformation virus (ToLDeV) DNA, (**E**) beet curly top virus (BCTV) DNA, and (**F**) sweet potato leaf curl virus (SPLCV) DNA in *N. benthamiana*. Data (2^−ΔΔCT^, Table S6) correspond to plants agroinoculated in two independent experiments (Exp.1, Exp.2) analyzed at 30 days post-inoculation. For SPLCV, included as a control, only Experiment 1 was analyzed. Each circle (DNA/DNA-A) and square (DNA-B) represents one infected plant. Open circles and squares correspond to outlier values. Mean and standard error values are indicated in each graph. Significant differences are labelled with asterisks (n.s., *p* > 0.05; *, *p* ≤ 0.05; **, *p* ≤ 0.01; ***, *p* ≤ 0.001; ****, *p* ≤ 0.0001). Additional details of the statistical analysis are provided in Table S7.

The accumulation of geminivirids acting as helper for the replication of SPLCD1 was determined by relative quantification with real-time PCR in agroinoculated plants in the presence or absence of the deltasatellite at 30 days post-inoculation (Table S6, Figure 7). Genome quantification for ToLCNDV, SiGYVV, ToLDeV, BCTV, and SPLCV (used as a control) was determined in *N. benthamiana* plants and for ToLCNDV also in zucchini plants. For ToLCNDV and SiGYVV, the accumulation of both DNA-A and DNA-B genome components was determined separately.

In *N. benthamiana* plants, accumulation of both DNA-A and DNA-B genome components of ToLCNDV increased in the presence of SPLCD1 in the two experiments performed (Figure 7A). On the contrary, in the case of SiGYVV, accumulation of both DNA-A and DNA-B decreased in the presence of SPLCD1 in both experiments (Figure 7C). Similarly, the accumulation of the ToLDeV genome also decreased in the presence of SPLCD1 in both experiments (Figure 7D). In the case of the curtovirus BCTV, no effect of the presence of SPLCD1 on viral genome accumulation was observed in either of the two experiments (Figure 7E). In the only experiment analyzed for SPLCV, used as a control, the negative effect of the deltasatellite on viral genome accumulation previously described [26] was confirmed (Figure 7F). Interestingly, in contrast to what was observed in *N. benthamiana* plants, the presence of SPLCD1 did not influence the accumulation of ToLCNDV DNA-A or DNA-B in zucchini plants in any of the two experiments performed (Figure 7B). A summary of the statistical analysis results is presented in Table S7.

## 4. Discussion

Deltasatellites are ssDNA molecules associated with begomoviruses belonging to different phylogenetic lineages including Old World and New World begomoviruses and sweepoviruses, being unique in that they are non-coding in contrast with alphasatellites and betasatellites [20,41,42]. Deltasatellites have been found in scattered regions around the world including the Americas, Europe, Asia, and Australia [19,20,23,24,25,41], but little is known about their genetic diversity and role in diseases caused by begomoviruses.

In this work the diversity of deltasatellites and their natural helper sweepoviruses infecting *I. indica* plants was analyzed by further sampling in Málaga province, the region where SPLCD1 was detected for the first time [19], expanding the sampling to other geographical areas of the coastal zone of southern continental Spain and the Canary Islands. RCA methodology, which allows ssDNA amplification without previous knowledge of nucleotide sequence, has been used to reveal the actual variability present in natural populations. Sequencing of a high number of deltasatellite full-length genomes from continental Spain (92 isolates from 46 samples) revealed a rather homogeneous population with low genetic diversity, which in addition did not seem to be geographically structured. None of the samples from the Canary Islands of Tenerife or Gran Canaria were infected by sweepovirus, a somehow surprising result considering that sweepoviruses infecting sweet potato were previously characterized from Tenerife [5]. In another of the Canary Islands, Lanzarote, sweepoviruses and deltasatellites have also been identified in sweet potato [5,19].

This study also gave insight into the nature of the helper sweepoviruses able to transreplicate deltasatellites, specifically SPLCD1. Most of the sweepovirus isolates to which SPLCD1 was found associated in *I. indica* plants belonged to the species *Sweet potato leaf curl virus*. Interestingly, sweepoviruses identified in three samples from Málaga and all three samples from Cádiz occupied a phylogenetic position intermediate between SPLCV and SPMV isolates. SPMV isolates have been previously reported only from Brazil and South Africa [8,13]. Pairwise comparisons strongly suggested that the species *Sweet potato mosaic virus* should merge with *Sweet potato leaf curl virus* but defining a distinct SPLCV strain should include isolates previously identified as SPMV and the six abovementioned Spanish isolates. Five out of these six sweepovirus isolates were found in co-infection with SPLCD1, thus expanding the sweepovirus range to which this deltasatellite is associated in nature.

Recombination between begomoviruses is frequent and contributes significantly to viral diversity, speciation, and evolution (e.g., [43,44]). The significance of this phenomenon has been well illustrated for sweepoviruses [5,45]. Recombination events have been identified in all sweepovirus genomes described in this work, including those recombinants that revealed that isolates previously described as SPMV should be considered members of a novel strain of *Sweet potato leaf curl virus*. Deltasatellites described in this work, in their turn, did now show any evidence of recombination between them or with deltasatellites previously described, including representatives of all accepted deltasatellite species.

What has been identified in this work is a recombination event that must have led to the formation of a deltasatellite-sweepovirus chimera with the typical size of a deltasatellite. The putative parentals involved in the generation of this chimeric molecule by recombination were isolates of SPLCV and SPLCD1 closely related to isolates present in the same *I. indica* sample (MW574045 and MW587238). A chimera also containing sweepovirus and deltasatellite sequences was found previously in a sweet potato plant sampled in the Canary Islands [19]. This chimera had the typical size of a sweepovirus, and about 70% of its length had high nucleotide identity with an isolate of sweet potato leaf curl Canary virus (V2, CP, and truncated REn genes plus complete IR) cloned from the same sample. The remaining chimera corresponded to almost the full-length sequence of a SPLCD1 genome. Interestingly, in both chimeras one of the recombination points contained an incomplete stem-loop derived from the deltasatellite. In geminivirids, the conserved stem-loop has been identified as a recombination hotspot [46,47]. Vegetative propagation is the method of choice for sweet potato, *I. indica*, and other ornamental species of the genus *Ipomoea*. This favors virus accumulation and perpetuation, mixed infections, and occurrence of recombination [5,45,48]. This phenomenon may result in the rapid generation of new genomes with adaptive advantages, which could accelerate their evolution and favor the expansion of the host range and, therefore, the emergence of novel diseases (e.g., [44,49,50]). The same mechanism that originates viral recombinants could generate the virus-deltasatellite chimera found in this study.

Phylogenetic analysis of a number of deltasatellites associated with NW begomoviruses in Cuba [20] or sweepoviruses in Spain [19] have revealed some clustering related to geographical origin and plant host. However, in the present study, no clear grouping of deltasatellites was observed related to the geographical origin of the *I. indica* samples.

In order to deepen the understanding of the role that deltasatellites may have on begomovirus diseases and epidemiology, the helper virus range of SPLCD1 was studied experimentally. Previous to this work, the experimental helper virus range of SPLCD1, in addition to SPLCV, was limited to two monopartite OW begomoviruses, TYLCV and TYLCSV [26]. In this work, using *N. benthamiana* as a plant host, the helper virus range was successfully extended to a bipartite OW begomovirus, ToLCNDV; a bipartite NW begomovirus, SiGYVV; a monopartite NW begomovirus, ToLDeV; and a curtovirus, BCTV. These compelling results indicate that SPLCD1 has a broad range of helper viruses including members of all major groups of begomoviruses and extending to members of a different virus genus in the family, the genus *Curtovirus*. This helper virus range is wider than that of other deltasatellites for which this has been studied in *N. benthamiana*: Sida golden yellow vein deltasatellite 1 (SiGYVD1) and tomato yellow leaf distortion deltasatellite 2 (ToYLDD2), deltasatellites naturally associated with bipartite NW begomoviruses. SiGYVD1 and ToYLDD2 were maintained by the monopartite NW begomovirus ToLDeV, in addition to their respective natural helper begomoviruses, but not by the monopartite OW begomoviruses ACMV, TYLCV, and TYLCSV or the curtovirus BCTV [34]. On the other hand, the first deltasatellite to be described, ToLCD, naturally associated with the monopartite OW begomovirus tomato leaf curl virus, was reported to be experimentally transreplicated in *Datura stramonium* plants by TYLCV, the bipartite OW begomovirus African cassava mosaic virus, and BCTV [41]. There is no information available about maintenance of ToLCD by NW begomoviruses or sweepoviruses.

In this work, the transreplication of SPLCD1 by ToLCNDV, SiGYVV, ToLDeV, and BCTV was also assessed in their natural host plants. The only positive result was obtained with ToLCNDV in zucchini, with about half of the virus-infected plants also infected by the deltasatellite. The fact that SPLCD1 can be transreplicated by ToLCNDV in zucchini may have significant epidemiological importance. This virus has a wide host range, infecting more than 40 plant species [51], and although the primary host is tomato, it also infects other economically important crops such as potato, pepper, and cucurbits. In fact, the isolate used in this work belongs to a strain introduced in the Mediterranean basin, very probably from the Indian subcontinent, adapted to cucurbits [33,52]. Considering the wide host range of ToLCNDV and its ability to transreplicate SPLCD1 at least in zucchini, as well as the transmissibility of the deltasatellite by whiteflies [26], co-infections involving ToLCNDV and SPLCD1 could provide an opportunity for this complex to expand to other crops and geographical regions where it could have unpredictable consequences.

Previous to this work, the influence of deltasatellites on helper virus accumulation had not been thoroughly addressed, with only a few analyses done by quantifying densitometry of Southern blots for a number of deltasatellite/begomovirus combinations. Summarizing, in most of the deltasatellite/begomovirus combinations agroinoculated in *N. benthamiana* plants (SPLCD1/SPLCV, SPLCD1/TYLCV, SPLCD1/TYLCSV, SiGYVD1/SiGYVV, and ToYLDD2/ToYLDV), the begomovirus accumulation decreased in the presence of the deltasatellite [26,34]. In contrast, no effect on virus accumulation was observed when other plant hosts were agroinoculated, including the combinations SPLCD1/SPLCV/*I. setosa*, SPLCD/TYLCV/tomato, SiGYVD1/SiGYVV/*M. coromandelianum*, and ToYLDD2/ToYLDV/*Sidastrum micranthum* [26,34]. In the case of the combinations SiGYVD1/ToLDeV and ToYLDD2/ToLDeV in *N. benthamiana*, no effect of the deltasatellite on viral accumulation was observed either [34]. In the present work, real-time PCR was used as a more accurate proxy for viral genome quantification. In the case of SPLCD1/SiGYVV and SPLCD1/ToLDeV in *N. benthamiana*, the presence of the deltasatellite decreased the begomovirus accumulation as it has been shown for most of the abovementioned cases. On the contrary, accumulation of the curtovirus BCTV was not affected by the presence of the deltasatellite.

A particular and interesting case is the response of ToLCNDV to the presence of SPLCD1. This is the first time that a deltasatellite has been shown to increase the accumulation of a helper geminivirid, in this case in *N. benthamiana* plants. On the other hand, this effect was not observed in zucchini plants, a natural host for the cucurbit-adapted ToLCNDV isolate used in this study [33], where SPLCD1 did not affect virus accumulation. The results of virus quantification obtained in this study, including those for ToLCNDV, were consistent in the two independent experiments performed for each deltasatellite/geminivirid combination and for both genome components (DNA-A, DNA-B) in the case of bipartite begomoviruses. This adds robustness to the otherwise surprising results obtained for ToLCNDV that would reveal the complexity of the deltasatellite/geminivirid/plant host interactions.

The contrasting effect of deltasatellites on helper virus accumulation depending on the virus–host combinations found in this study and in previous research [26,34], exemplified in the case of SPLCD1/ToLCNDV combination, has been described for other DNA satellites associated with begomoviruses. Thus, the accumulation of Euphorbia yellow mosaic virus (EuYMV) DNA-A increases in the presence of Euphorbia yellow mosaic alphasatellite (EuYMA) in two plant hosts, *Euphorbia heterophylla* and *N. benthamiana* [53]. However, the presence of EuYMA causes a reduction in the accumulation of EuYMV DNA-A in *Arabidopsis thaliana*. For EuYMV DNA-B, no differences in its accumulation are observed in the presence or absence of EuYMA in both *N. benthamiana* and *A. thaliana*, but results in *E. heterophylla* show an increase of EuYMV DNA-B accumulation in the presence of EuYMA [53].

In most cases where a possible effect of deltasatellites on the symptomatology caused by the helper geminivirid has been assessed, no symptom modifications have been observed, despite the effect on virus accumulation that was observed for some of the deltasatellite/virus combinations [26,34,41]. The only exception is the effect of SPLCD1 on the symptoms caused by TYLCV and TYLCSV in *N. benthamiana* or tomato [26]. In this case, although the symptoms were qualitatively identical in the absence or presence of the deltasatellite, in the latter case milder leaf yellowing and curling was observed.

In nature, known deltasatellites seem to have a narrow helper virus range because closely related isolates of a single begomovirus species have been reported per deltasatellite species [19,20,23,34,41]. However, in the present study SPLCD1 was found associated with two distinct SPLCV strains, suggesting that a somewhat broader helper virus range could occur naturally. This situation is different from what is observed for betasatellites that are frequently found associated with begomovirus isolates belonging to different species [42].

A narrow helper virus range of deltasatellites in turn would restrict the plant host range that deltasatellites could potentially infect. However, the promiscuous replicative nature of deltasatellites that is being revealed in this and other studies [26,34,41], coupled with global trade and whitefly transmission, could facilitate dissemination of deltasatellites to diverse agrosystems with unforeseeable outcomes.

Although deltasatellites and betasatellites are clearly related [19], there are fundamental differences between them, including the non-coding nature of the formers. This feature hinders trying to address the identification of deltasatellite motifs/sequences involved in the interaction with their helper viruses and pathogenesis. Anyhow, mutation studies similar to those successfully performed with betasatellites [54,55] could help to decipher how deltasatellites are able to be transreplicated by different helper geminivirids and whether any of the hypotheses proposed for betasatellite-begomovirus recognition, the “universal Rep” hypothesis or the “universal iteron” hypothesis” [56], could also be applied to these small non-coding DNA molecules.

## Figures and Tables

**Table 1:** *Ipomoea indica* samples analyzed in this study. Additional details are given in Table S1.

Province/ Island	Number of Samples	Number of Symptomatic Samples		Number of Infected Samples	
		Yellow Veins (%)	Leaf Curling (%)	Sweepoviruses (%)	Deltasatellites (%)
Murcia	5	1 (20.0)	1 (20.0)	5 (100.0)	1 (20.0)
Granada	5	0 (0.0)	0 (0.0)	5 (100.0)	5 (100.0)
Málaga	52	5 (9.6)	5 (9.6)	46 (88.5)	38 (73.1)
Cádiz	3	0 (0.0)	0 (0.0)	3 (100.0)	2 (66.7)
Tenerife	12	3 (25.0)	3 (25.0)	0 (0.0)	0 (0.0)
Gran Canaria	12	8 (66.7)	7 (58.3)	0 (0.0)	0 (0.0)
Continental Spain ^1^	65	6 (9.2)	6 (9.2)	59 (90.8)	46 (70.8)
Canary Islands ^2^	24	11 (45.8)	10 (41.7)	0 (0.0)	0 (0.0)
Total	89	17 (19.1)	16 (18.0)	59 (66.3)	46 (51.7)

^1^ Murcia, Granada, Málaga, and Cádiz provinces. ^2^ Tenerife and Gran Canaria islands.

**Table 2:** Infectivity of sweet potato leaf curl deltasatellite 1 (SPLCD1) in the presence of various geminivirids in *Nicotiana benthamiana* plants.

Virus (+Deltasatellite)	Number of Infected Plants/Number of Agroinoculated Plants				Total (%)	
	Experiment 1		Experiment 2			
	Virus	SPLCD1	Virus	SPLCD1	Virus	SPLCD1
ToLCNDV	14/15	0/15	15/15	0/15	96.7	0.0
ToLCNDV + SPLCD1	15/15	15/15	15/15	15/15	100.0	100.0
SiGYVV	15/15	0/15	15/15	0/15	100.0	0.0
SiGYVV + SPLCD1	15/15	15/15	15/15	15/15	100.0	100.0
ToLDeV	15/15	0/15	15/15	0/15	100.0	0.0
ToLDeV + SPLCD1	15/15	15/15	15/15	15/15	100.0	100.0
BCTV	15/15	0/15	15/15	0/15	100.0	0.0
BCTV + SPLCD1	15/15	7/15	15/15	8/15	100.0	50.0
SPLCV (positive control)	12/12	0/12	12/12	0/12	100.0	0.0
SPLCV + SPLCD1 (positive control)	12/12	12/12	12/15	12/12	100.0	100.0
Mock (negative control)	0/15	0/15	0/15	0/15	0.0	0.0

ToLCNDV, tomato leaf curl New Delhi virus; SiGYVV, Sida golden yellow vein virus; ToLDeV, tomato leaf deformation virus; BCTV, beet curly top virus; SPLCV, sweet potato leaf curl virus.

**Table 3:** Infectivity of sweet potato leaf curl deltasatellite 1 (SPLCD1) in the presence of various geminivirids in zucchini, *Malvastrum coromandelianum*, and tomato plants.

Plant Host	Virus (+Deltasatellite)	Number of Infected Plants/Number of Agroinoculated Plants						Total (%)	
		Experiment 1		Experiment 2		Experiment 3			
		Virus	SPLCD1	Virus	SPLCD1	Virus	SPLCD1	Virus	SPLCD1
Zucchini	ToLCNDV	15/15	0/15	15/15	0/15	–	–	100.0	0.0
	ToLCNDV + SPLCD1	15/15	5/15	14/15	9/15	–	–	96.6	46.6
	Mock (negative control)	0/15	0/15	0/15	0/15	–	–	0.0	0.0
*M. c.*	SiGYVV	17/32	0/32	16/32	0/32	–	–	51.5	0.0
	SiGYVV + SPLCD1	16/32	0/32	15/32	0/32	–	–	48.4	0.0
	Mock (negative control)	0/15	0/15	0/15	0/15	–	–	0.0	0.0
Tomato	ToLDeV	15/15	0/15	60/60	0/60	48/48	0/48	100.0	0.0
	ToLDeV + SPLCD1	14/15	0/15	55/60	0/60	103/108	0/108	93.9	0.0
	BCTV	15/15	0/15	66/66	0/66	–	–	100.0	0.0
	BCTV + SPLCD1	15/15	0/15	57/66	0/66	–	–	88.8	0.0
	Mock (negative control)	0/15	0/15	0/15	0/15	0/15	0/15	0.0	0.0

*M. c.*, Malvastrum coromandelianum; –, not done.

## Data Availability

All nucleotide sequences obtained in this study have been deposited in GenBank under accession numbers MW574018-MW574052 for sweepovirus genomes, MW587160-MW587196 and MW587198-MW587252 for deltasatellite genomes, and MW587197 for the sweepovirus-deltasatellite chimera. Data of real-time PCR (2−ΔΔCt values) are provided as supplementary material.

## Supplementary Materials

The following are available online at https://www.mdpi.com/article/10.3390/microorganisms9051018/s1, Figure S1: Color-coded matrix of pairwise sequence identity scores generated by alignment of the full-length genomes of the sweepoviruses obtained in this work, Figure S2: Recombination analysis using RDP4 package of the sweepovirus genomes obtained in this work, Figure S3: Color-coded matrix of pairwise sequence identity scores generated by alignment of the full-length genomes of the deltasatellites obtained in this work, Figure S4: DNA hybridization of petiole cross section blots of newly emerged young leaves of *Nicotiana benthamiana* plants agroinoculated with ToLCNDV, SiGYVV, ToLDeV, or BCTV alone or in combination with SPLCD1, Figure S5: DNA hybridization of petiole cross section blots of newly emerged young leaves of zucchini plants agroinoculated with ToLCNDV and SPLCD1, Table S1: Information on the *Ipomoea indica* samples used in this study, Table S2: Sweepovirus sequences retrived from GenBank used for pairwise sequence identity, phylogenetic, and recombination analyses, Table S3: Deltasatellite sequences retrieved from GenBank used for pairwise sequence identity and phylogenetic and recombination analyses, Table S4: List of primers used by real-time PCR to amplify the deltasatellite and viral DNA or plant reference genes, Table S5: Estimate of the coefficient of evolutionary differentiation for SPLCV and SPLCD1 genomes obtained in this work and isolates previously reported from Spain, Table S6: Data of virus quantification by real-time PCR (2^−ΔΔCt^) used to generate Figure 7 with Graphpad Prism 6.0 software, Table S7: Summary of statistical analyses performed to evaluate the effect of SPLCD1 on accumulation of helper geminivirids.
