# Supplementary material for: Revealing the Complexity of Sweepovirus-Deltasatellite–Plant Host Interactions: Expanded Natural and Experimental Helper Virus Range and Effect Dependence on Virus-Host Combination
Source: Microorganisms. 2021 May 10;9(5):1018. doi: 10.3390/microorganisms9051018 (PMC8150397; doi:10.3390/microorganisms9051018)
Supplement: Supplementary file 1 [file microorganisms-09-01018-s001.zip › microorganisms-1195267-supplementary.pdf]

Revealing the Complexity of Sweepovirus-Deltasatellite-Plant Host Interactions: Expanded Natural and Experimental Helper Virus Range and Effect Dependence on Virus-Host Combination

Camila G. Ferro, F. Murilo Zerbini, Jesús Navas-Castillo and Elvira Fiallo-Olivé

Supplementary Materials

Figure S1: Color-coded matrix of pairwise sequence identity scores generated by alignment of the full-length genomes of the sweepoviruses obtained in this work.

Figure S2: Recombination analysis using RDP4 package of the sweepovirus genomes obtained in this work.

Figure S3: Color-coded matrix of pairwise sequence identity scores generated by alignment of the full-length genomes of the deltasatellites obtained in this work.

Figure S4: DNA hybridization of petiole cross section blots of newly emerged young leaves of *Nicotiana benthamiana* plants agroinoculated with ToLCNDV, SiGYVV, ToLDeV or BCTV alone or in combination with SPLCD1.

Figure S5: DNA hybridization of petiole cross section blots of newly emerged young leaves of zucchini plants agroinoculated with ToLCNDV and SPLCD1.

Table S1: Information of the *Ipomoea indica* samples used in this study.

Table S2: Sweepovirus sequences retrived from GenBank used for pairwise sequence identity, phylogenetic and recombination analyses.

Table S3: Deltasatellite sequences retrieved from GenBank used for pairwise sequence identity, phylogenetic and recombination analyses.

Table S4: List of primers used for real-time PCR to amplify the deltasatellite and viral DNA or plant reference genes.

Table S5: Estimate of the coefficient of evolutionary differentiation for SPLCV and SPLCD1 genomes obtained in this work and isolates previously reported from Spain.

Table S6: Data of virus quantification by real-time PCR ($2^{-\Delta\Delta Ct}$) used to generate Figure 7 with Graphpad Prism 6.0 software.

Table S7: Summary of statistical analyses performed to evaluate the effect of SPLCD1 on accumulation of helper geminivirids.

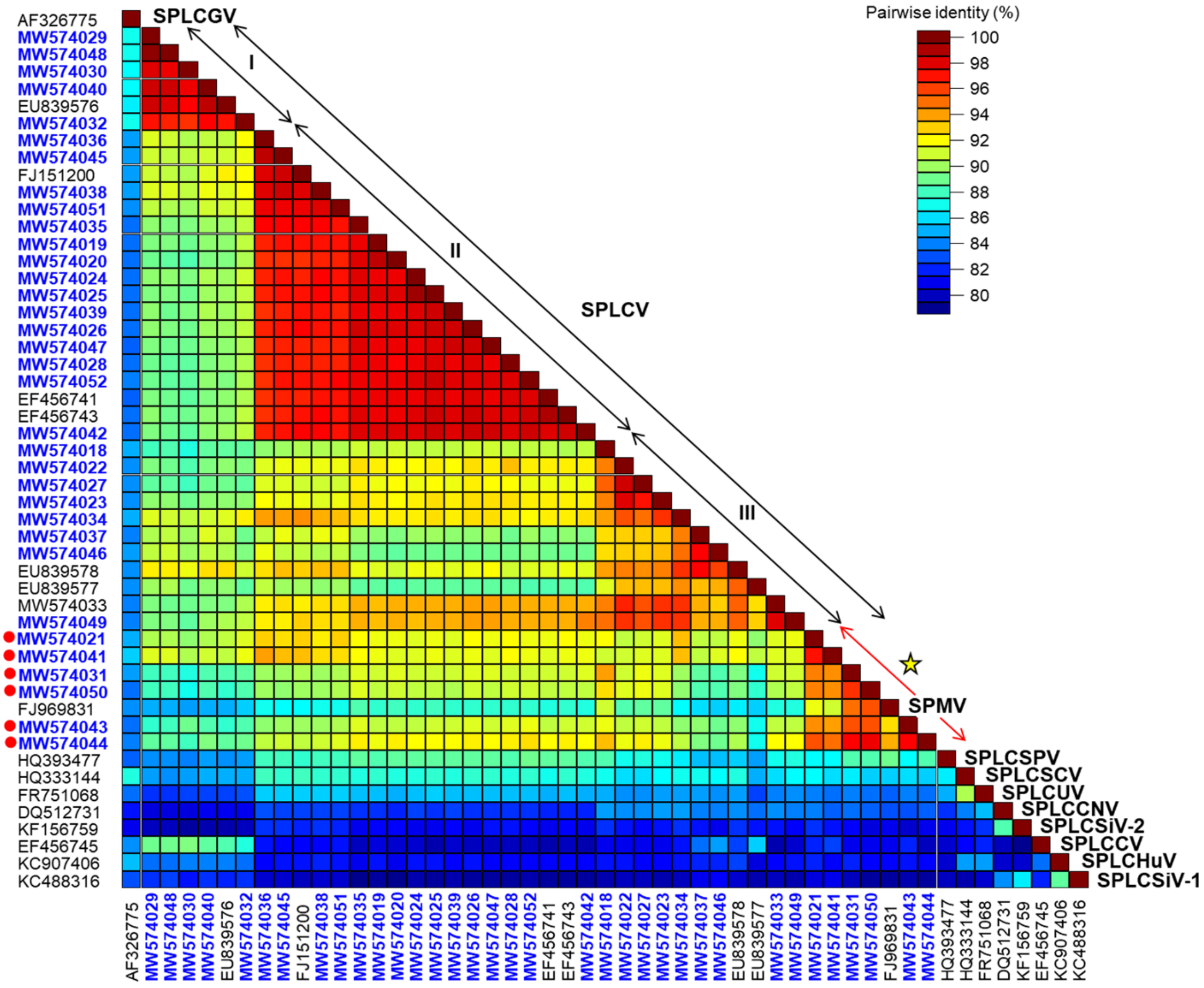

Figure S1. Color-coded matrix of pairwise sequence identity scores generated by alignment of the full-length genomes of the sweepoviruses obtained in this work (highlighted in blue), closely related sweet potato leaf curl virus (SPLCV) isolates previously reported from Spain and one representative isolate of all other sweepovirus species. I to III represent subgroups within the group of SPLCV isolates. The yellow star marks the group of sweepovirus sequences described in this work (red dots) showing ≥91% nucleotide identity with both sweet potato mosaic virus and SPLCV isolates. Additional details on the sequences and sweepovirus names are included in Tables S1 and S2.

A

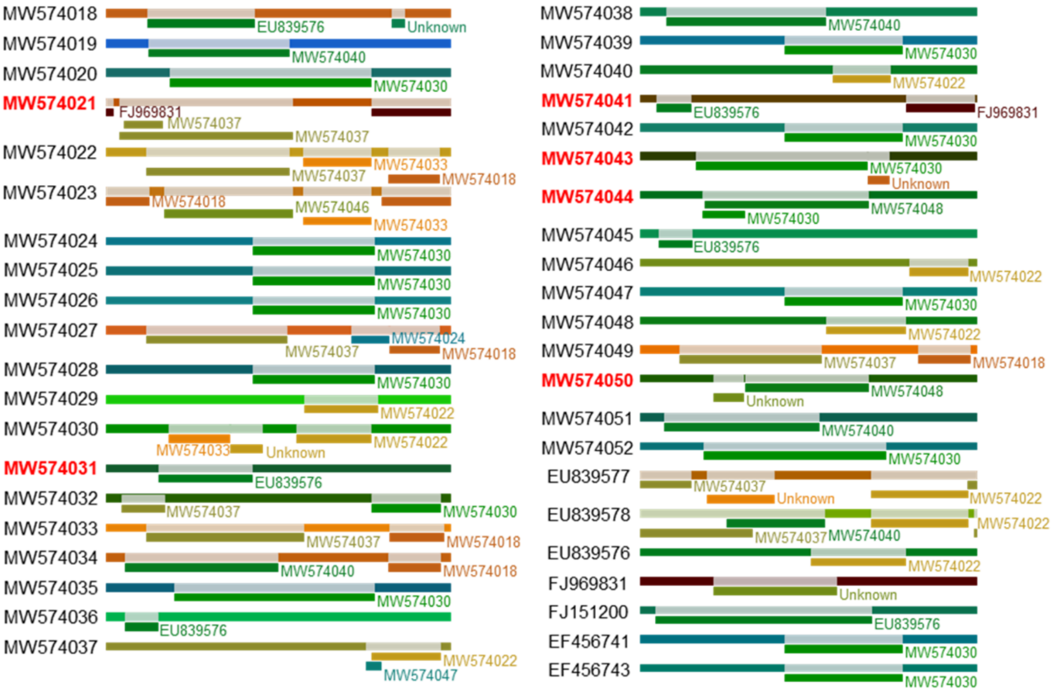

B

Event no.	Found in	Recombinant	Major parent	Minor parent	R G B M C S T
1	4	MW574046	MW574048	MW574022	+ + + + – + +
2	1	**MW574021**	MW574032	FJ969831	+ + + + + + +
3	1	**MW574041**	MW574032	FJ969831	+ + + + + + +
4	2	**MW574050**	FJ969831	MW574048	+ + + + + + +
5	4	FJ151200	MW574039	EU839576	+ + + + + + +
6	14	**MW574043**	FJ969831	MW574030	+ + + + + + +
7	6	MW574034	MW574032	MW574018	+ + + + + + +
8	2	EU839577	MW574022	MW574037	+ + + + + + +
9	5	MW574030	MW574037	MW574022	+ + + + + + +
10	1	MW574032	MW574038	MW574030	+ + + + – + +
11	1	MW574018	FJ969831	EU839576	+ + + + + + +
12	1	**MW574031**	FJ969831	EU839576	+ + + + + + +
13	5	MW574038	FJ969831	MW574040	+ + + + + + +
14	2	MW574032	MW574033	MW574037	+ + + + + + +
15	1	EU839577	MW574046	Unknown	+ + + + + + +
16	2	FJ969831	MW574027	Unknown	+ + + + + + +
17	1	MW574030	MW574029	Unknown	+ + + + + – +
18	2	MW574023	Unknown	MW574033	+ + + + + + +
19	1	**MW574043**	**MW574031**	Unknown	+ + + + + – +
20	1	MW574023	MW574027	MW574046	+ – – + + – +
21	1	MW574030	MW574029	MW574033	+ + + + + + +
22	1	MW574037	MW574046	MW574047	– + + + + + +
23	5	MW574027	MW574018	MW574037	+ + + + + + +
24	1	MW574027	MW574018	MW574024	+ + + + + + +
25	1	MW574018	**MW574031**	Unknown	+ + – + + – +

Figure S2. Recombination analysis using RDP4 package [32] of the sweepovirus genomes obtained in this work (MW574018-MW574052), closely related sweet potato leaf curl virus (SPLCV) isolates previously reported from Spain and a sweet potato mosaic virus (SPMV) isolate (FJ969831). (**A**) Schematic diagram showing putative recombinant fragments. (**B**) Overview of the recombination results showing for each recombination event the number of sequences where it was found, the putative recombinant sequence, the major and minor parents, and the methods (R, RDP; G, GENECONV; B, BOOTSCAN; M, MAXIMUM CHI SQUARE; C, CHIMAERA; S, SISTER SCAN and 3S, 3SEQ) that identified (+) it. Details on sweepovirus sequences retrieved from GenBank are given in Table S2. GenBank accessions numbers for sweepovirus isolates occupying an intermediate position between SPLCV and SPMV isolates are highlighted in red.

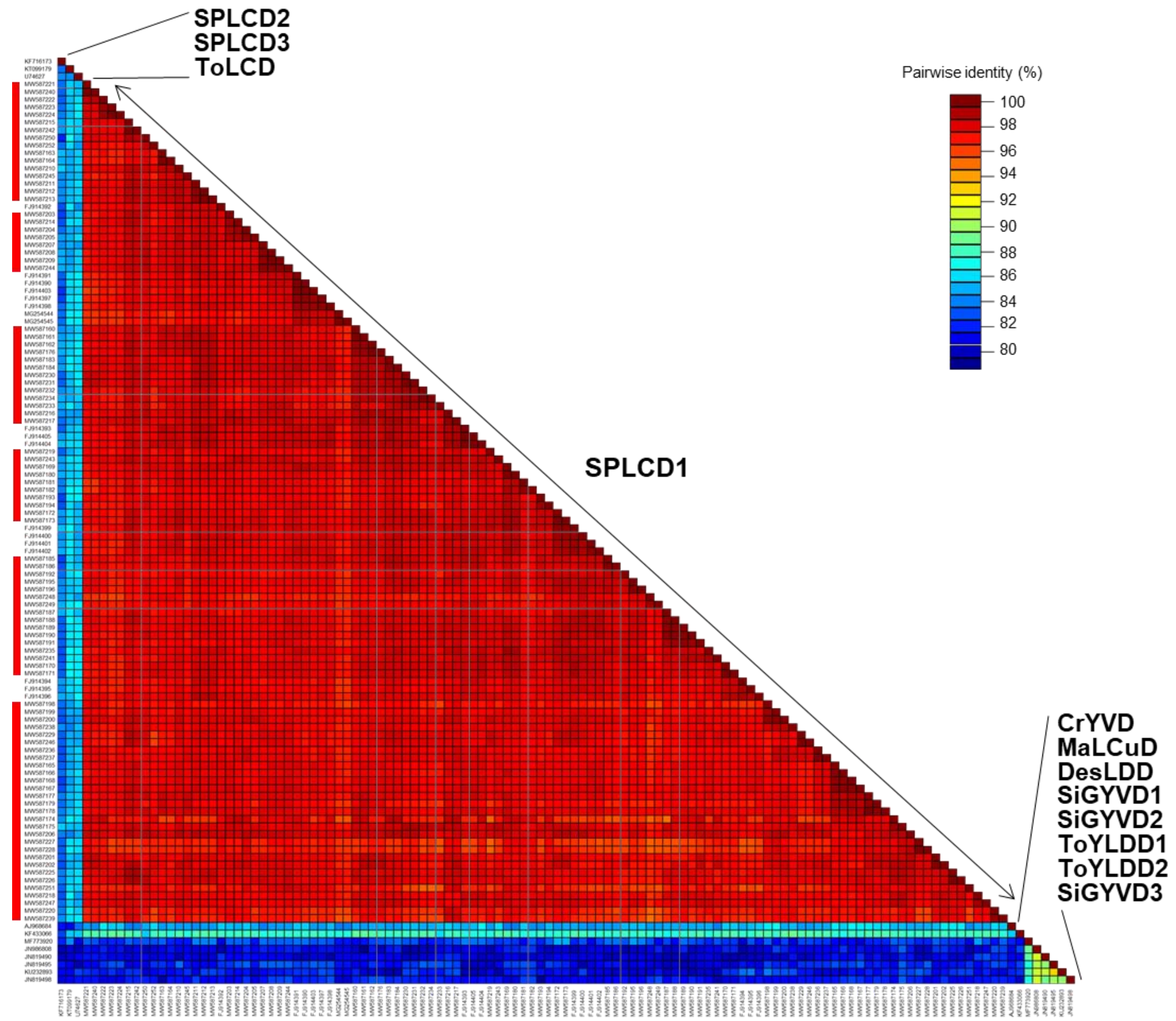

Figure S3. Color-coded matrix of pairwise sequence identity scores generated by alignment of the full-length genomes of the deltasatellites obtained in this work (marked with red lines at the left of the graph), sweet potato leaf curl deltasatellite 1 (SPLCD1) isolates previously reported from Spain and Portugal and one representative isolate of all other deltasatellite species. Additional details on the sequences and deltasatellite names are included in Tables S1 and S3.

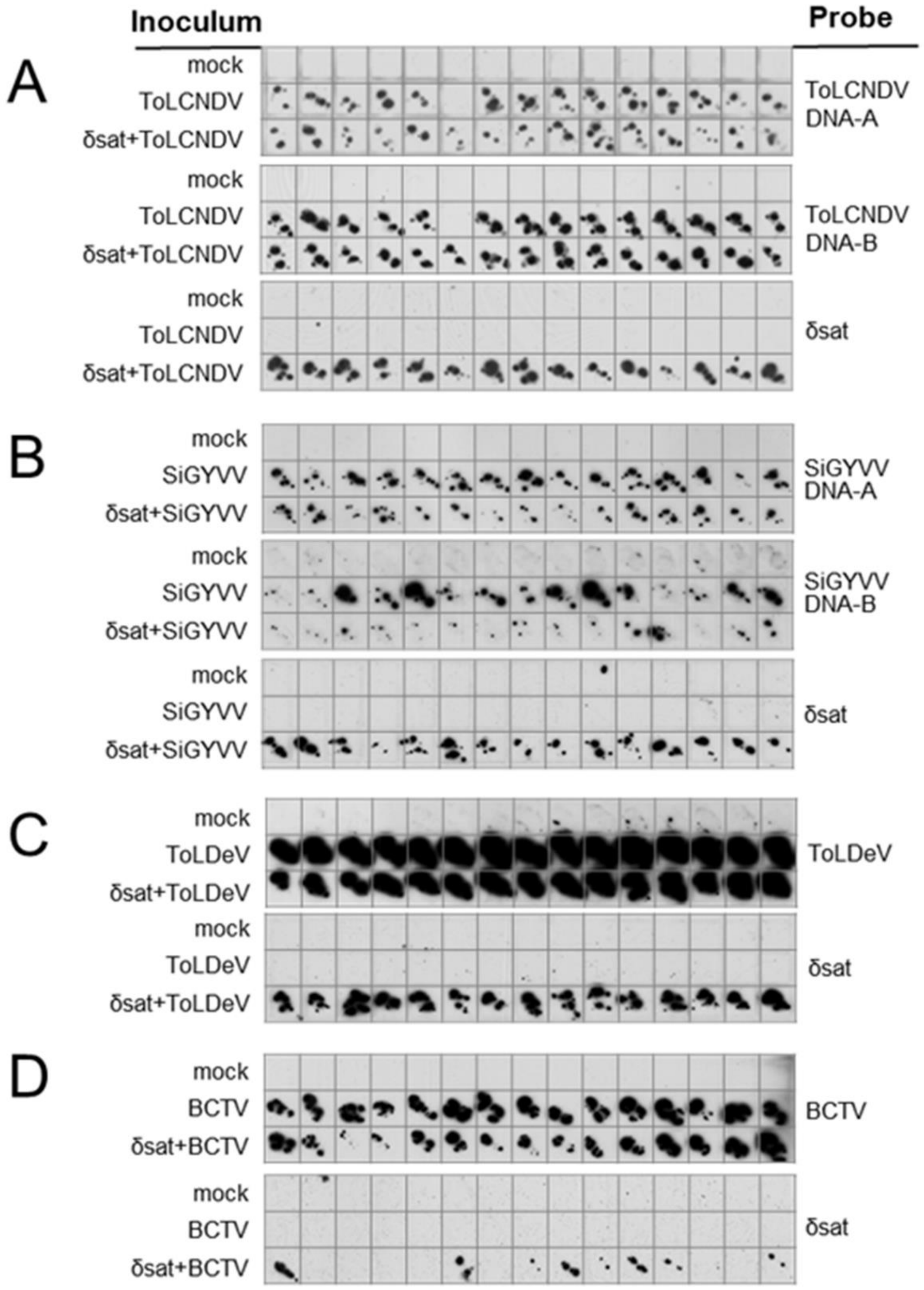

Figure S4. DNA hybridization of petiole cross section blots of newly emerged young leaves of *Nicotiana benthamiana* plants agroinoculated with (**A**) tomato leaf curl New Delhi virus (ToLCNDV), (**B**) Sida golden yellow vein virus (SiGYVV), (**C**) tomato leaf deformation virus (ToLDeV) or (**D**) beet curly top virus (BCTV) alone or in combination with sweet potato leaf curl deltasatellite 1 (δsat). Probes used are indicated at the right of the figure. One of two experiments carried out with each geminivirid-deltasatellite combination (Table 2) is shown.

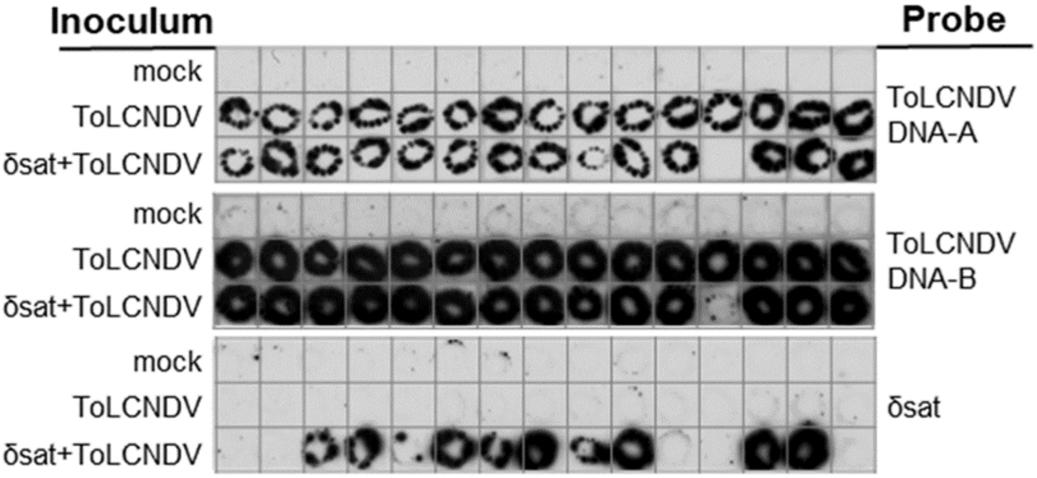

Figure S5. DNA hybridization of petiole cross section blots of newly emerged young leaves of zucchini plants agroinoculated with tomato leaf curl New Delhi virus (ToLCNDV) and sweet potato leaf curl deltasatellite 1 (δsat). Probes used are indicated at the right of the figure. Experiment 2 (Table 3) is shown.

Table S1. Information of the *Ipomoea indica* samples used in this study.

Province/ island	Sample code	Sampling date	Geographical coordinates	Symptoms	RCA analysis		Sweepovirus GenBank acc. no.	Deltasatellite GenBank acc. no.
					Sweepo-virus	Delta-satellite		
Murcia	ii59	Oct-15	37°55.582′N 1°07.544′W	-	+	-	-	-
	ii60	Oct-15	38°03.130′N 1°13.032′W	-	+	-	-	-
	ii61	Oct-15	38°03.130′N 1°13.032′W	LC	+	+	MW574052	MW587248 MW587249
	ii62	Oct-15	38°03.130′N 1°13.032′W	YV	+	-	-	-
	ii63	Oct-15	38°03.130′N 1°13.032′W	-	+	-	-	-
Granada	ii49	Aug-15	36°44.182′N 3°40.806′W	-	+	+	-	MW587206
	ii50	Aug-15	36°44.612′N 3°33.341′W	-	+	+	MW574051	MW587242 MW587215
	ii51	Aug-15	36°43.562′N 3°32.320′W	-	+	+	MW574042	MW587243
	ii52	Aug-15	36°44.037′N 3°32.930′W	-	+	+	-	MW587244
	ii53	Aug-15	36°44.041′N 3°32.925′W	-	+	+	MW574049	MW587207 MW587208 MW587209
Málaga	ii01	Feb-15	36°45.444′N 4°02.654′W	-	+	-	-	-
	ii02	Feb-15	36°45.452′N 4°02.670′W	-	+	-	-	-
	ii03	Feb-15	36°45.453′N 4°02.687′W	-	+	+	MW574018	MW587174 MW587175
	ii04	Feb-15	36°44.794′N 4°03.014′W	-	+	-	-	-
	ii05	Feb-15	36°44.800′N 4°03.013′W	-	+	-	-	-
	ii06	Feb-15	36°44.053′N 4°06.920′W	-	+	+	MW574019	MW587160 MW587161
	ii07	Feb-15	36°44.041′N 4°06.927′W	YV, LC	+	+	MW574020	MW587162 MW587176
	ii08	Feb-15	36°44.084′N 4°06.930′W	-	+	+	MW574021	MW587163 MW587164
	ii09	Feb-15	36°46.002′N 4°06.455′W	YV	+	+	MW574027 MW574028	MW587165 MW587166
	ii10	Feb-15	36°45.994′N 4°06.460′W	YV, LC	+	+	MW574022	MW587167 MW587168
	ii11	Feb-15	36°45.990′N 4°06.463′W	-	+	+	MW574023	MW587177 MW587178 MW587179
	ii12	Feb-15	36°47.620′N 4°07.252′W	YV, LC	+	+	MW574024	MW587169 MW587180
	ii13	Feb-15	36°47.640′N 4°07.254′W	-	+	+	MW574025	MW587170 MW587171
	ii14	Feb-15	36°47.954′N 4°07.369′W	-	+	+	MW574026	MW587172 MW587173
	ii15	Feb-15	36°47.952′N 4°07.362′W	-	-	-	-	-
	ii16	Feb-15	36°49.881′N 4°07.555′W	-	-	-	-	-
	ii17	Feb-15	36°49.896′N 4°07.538′W	-	-	-	-	-
	ii18	May-15	36°44.748′N 4°02.216′W	-	+	+	-	MW587181 MW587182
	ii19	May-15	36°44.629′N 4°02.164′W	YV	+	+	-	MW587183 MW587184
	ii20	May-15	36°44.631′N 4°01.113′W	-	+	+	-	MW587185 MW587186
	ii21	May-15	36°43.967′N 3°56.509′W	LC	+	+	MW574029	MW587187 MW587188
	ii22	May-15	36°43.963′N 3°56.510′W	LC	+	+	MW574048	MW587189 MW587190 MW587191
	ii23	May-15	36°44.162′N 3°55.942′W	-	+	+	MW574030	MW587192 MW587193 MW587194
	ii24	May-15	36°44.189′N 3°55.878′W	-	-	-	-	-
	ii25	May-15	36°44.191′N 3°55.870′W	-	+	+	MW574033	MW587195 MW587196
	ii26	May-15	36°44.668′N 3°52.526′W	-	+	+	MW574045	MW587238
	ii27	May-15	36°44.660′N 3°52.539′W	-	+	+	MW574031	MW587198 MW587199 MW587200
	ii28	May-15	36°44.710′N 3°52.531′W	-	+	+	MW574034	MW587201 MW587202
	ii29	May-15	36°44.883′N 3°52.261′W	-	-	-	-	-
	ii30	May-15	36°44.877′N 3°52.248′W	-	+	+	MW574032	MW587216 MW587217
	ii31	May-15	36°44.880′N 3°52.259′W	-	+	+	MW574035	MW587247 MW587218
	ii32	May-15	36°45.466′N 3°51.732′W	-	+	+	-	MW587219
	ii33	May-15	36°45.468′N 3°51.750′W	-	+	+	-	MW587220 MW587239
	ii34	May-15	36°45.291′N 3°51.200′W	-	+	+	MW574036	MW587221 MW587240
	ii35	May-15	36°45.294′N 3°51.115′W	-	+	+	MW574046	MW587222 MW587223 MW587224
	ii36	May-15	36°44.448′N 3°45.639′W	-	+	+	-	MW587225 MW587226
	ii37	May-15	36°44.279′N 3°45.639′W	-	+	-	-	-
	ii38	May-15	36°44.374′N 3°41.753′W	-	+	+	MW574037	MW587227 MW587228
	ii39	May-15	36°44.367′N 3°41.736′W	-	+	+	-	MW587229 MW587246
	ii40	May-15	36°44.775′N 3°36.233′W	-	+	+	MW574038	MW587230 MW587231
	ii41	May-15	36°44.807′N 3°36.210′W	-	+	+	-	MW587232 MW587233 MW587234
	ii42	May-15	36°42.543′N 3°29.545′W	-	+	+	MW574039	MW587235 MW587241
	ii43	May-15	36°42.486′N 3°29.541′W	-	+	+	MW574040	MW587236 MW587237
	ii44	May-15	36°49.914′N 3°31.106′W	-	+	-	-	-
	ii45	Aug-15	36°45.728′N 3°53.302′W	-	+	+	-	MW587245
	ii46	Aug-15	36°45.692′N 3°53.302′W	-	-	-	-	-
	ii47	Aug-15	36°45.839′N 3°53.291′W	-	+	+	MW574041	MW587203 MW587214
	ii48	Aug-15	36°45.858′N 3°53.307′W	-	+	+	-	MW587204 MW587205
	ii54	Sep-15	36°27.268′N 5°4.862′W	-	+	-	-	-
	ii55	Sep-15	36°27.254′N 5°4.850′W	-	+	-	-	-
	ii64	Oct-15	36°44.686′N 4°5.275′W	-	+	+	MW574047	MW587250
	ii65	Oct-15	36°44.686′N 4°5.275′W	-	+	+	-	MW587251 MW587252
Cádiz	ii56	Sep-15	36°4.824′N 5°30.168′W	-	+	-	MW574043	-
	ii57	Sep-15	36°4.803′N 5°30.106′W	-	+	+	MW574050	MW587210 MW587211
	ii58	Sep-15	36°4.824′N 5°30.096′W	-	+	+	MW574044	MW587212 MW587213
Tenerife	ii66	Feb-15	28°30.814′N 16°23.159′W	-	-	-	-	-
	ii67	Feb-15	28°30.824′N 16°23.154′W	-	-	-	-	-
	ii68	Feb-15	28°34.066′N 16°19.707′W	-	-	-	-	-

	ii69	Feb-15	28°32.093′N 16°23.677′W	-	-	-	-	-
	ii70	Feb-15	28°34.372′N 16°11.228′W	-	-	-	-	-
	ii71	Feb-15	28°34.413′N 16°11.285′W	-	-	-	-	-
	ii72	Feb-15	28°28.555′N 16°19.996′W	YV, LC	-	-	-	-
	ii73	Feb-15	28°28.555′N 16°19.973′W	YV, LC	-	-	-	-
	ii74	Feb-15	28°28.555′N 16°19.965′W	YV, LC	-	-	-	-
	ii75	Feb-15	28°28.568′N 16°24.746′W	-	-	-	-	-
	ii76	Feb-15	28°28.574′N 16°24.769′W	-	-	-	-	-
	ii77	Feb-15	28°22.818′N 16°41.515′W	-	-	-	-	-
Gran Canaria	ii78	Feb-15	28°00.257′N 15°24.288′W	-	-	-	-	-
	ii79	Feb-15	28°02.584′N 15°28.636′W	YV	-	-	-	-
	ii80	Feb-15	28°02.606′N 15°28.631′W	YV, LC	-	-	-	-
	ii81	Feb-15	28°06.843′N 15°29.758′W	-	-	-	-	-
	ii82	Feb-15	28°01.535′N 15°30.281′W	YV, LC	-	-	-	-
	ii83	Feb-15	28°01.541′N 15°30.276′W	YV, LC	-	-	-	-
	ii84	Feb-15	28°01.335′N 15°30.663′W	-	-	-	-	-
	ii85	Feb-15	28°03.484′N 15°34.002′W	YV, LC	-	-	-	-
	ii86	Feb-15	28°03.785′N 15°33.182′W	YV, LC	-	-	-	-
	ii87	Feb-15	28°05.608′N 15°29.993′W	YV, LC	-	-		-
	ii88	Feb-15	28°05.599′N 15°30.004′W	YV, LC	-	-		-
	ii89	Feb-15	27°54.447′N 15°30.588′W	-	-	-		-

+, presence of sweepoviruses or deltasatellites; -, absence of symptoms, sweepoviruses, deltasatellites or nucleotide sequences; YV, yellow veins; LC, leaf curling.

Table S2. Sweepovirus sequences retrived from GenBank used for pairwise sequence identity, phylogenetic and recombination analyses.

**Acronym**	**Plant host**	**Location (country)**	**Genbank acc. no.**
SPLCCNV	Sweet potato	Liaoning (CN)	DQ512731
SPLCCV	Sweet potato	Canary Islands (ES)	EF456745
SPLCGV	Sweet potato	Georgia (US)	AF326775
SPLCHnV	Sweet potato	Henan (CN)	KC907406
SPLCSCV	Sweet potato	South Carolina (US)	HQ333144
SPLCSiV-1	Sweet potato	Sichuan (CN)	KC488316
SPLCSiV-2	Sweet potato	Sichuan (CN)	KF156759
SPLCSPV	Sweet potato	São Paulo (BR)	HQ393477
SPLCUV	Sweet potato	Kampala (UG)	FR751068
SPLCV	Sweet potato	Canary Islands (ES)	EF456741
	Sweet potato	Carnary Islands (ES)	EF456743
	Ipomoea indica	Málaga (ES)	FJ151200
	Ipomoea indica	Málaga (ES)	EU839576
	Ipomoea indica	Málaga (ES)	EU839577
	Ipomoea indica	Málaga (ES)	EU839578
SPMV	Sweet potato	Brasilia (BR)	FJ969831

SPLCCNV, sweet potato leaf curl China virus; SPLCCV, sweet potato leaf curl Canary virus; SPLCHnV, sweet potato leaf curl Henan virus; SPLCSiV-1, sweet potato leaf curl Sichuan virus 1; SPLCSiV-2, sweet potato leaf curl Sichuan virus 2; SPLCSPV, sweet potato leaf curl Sao Paulo virus; SPLCUV, sweet potato leaf curl Uganda virus; SPLCV, sweet potato leaf curl vírus; SPMV, sweet potato mosaic virus.

Table S3. Deltasatellite sequences retrieved from GenBank used for pairwise sequence identity, phylogenetic and recombination analyses.

Deltasatellite	Isolate	Host	Location (country)	GenBank acc. no.
CrYVD	IN-09	*Croton bonplandianus*	Madurai (IN)	AJ968684
DesLDD	CU-Co704-H1-13	*Corchorus siliquosus*	Matanzas (CU)	MF773920
MaLCuD	PH-12	*Malvastrum coromandelianum*	Philippines	KF433066
SiGYVD1	CU-177H1-09	*Malvastrum coromandelianum*	Camaguey (CU)	JN986808
SiGYVD2	CU-228H1-09	*Malvastrum coromandelianum*	Holguin (CU)	JN819490
SiGYVD3	CU-412N1-10	*Malvastrum coromandelianum*	Matanzas (CU)	JN819498
SPLCD1	ES-SBG32-02	Sweet potato	Canary Islands (ES)	FJ914391
	ES-SBG51-02	Sweet potato	Canary Islands (ES)	FJ914390
	ES-SBG57-02	Sweet potato	Canary Islands (ES)	FJ914397
	ES-SBG58-02	Sweet potato	Canary Islands (ES)	FJ914398
	ES-SBG59-02	Sweet potato	Canary Islands (ES)	FJ914403
	ES-SBG52-02	Sweet potato	Málaga (ES)	FJ914392
	ES-SBG54-02	Sweet potato	Málaga (ES)	FJ914394
	ES-SBG53-02	Sweet potato	Málaga (ES)	FJ914393
	ES-SBG55-02	Sweet potato	Málaga (ES)	FJ914395
	ES-SBG56-02	Sweet potato	Málaga (ES)	FJ914396
	ES-SBG3-5-02	Sweet potato	Málaga (ES)	FJ914404
	ES-SBGB3-6-02	Sweet potato	Málaga (ES)	FJ914405
	ES-SI3C-3-06	*Ipomoea indica*	Málaga (ES)	FJ914399
	ES-SI3C-5-06	*Ipomoea indica*	Málaga (ES)	FJ914400
	ES-SI3D-11-06	*Ipomoea indica*	Málaga (ES)	FJ914401
	ES-SI3D-12-06	*Ipomoea indica*	Málaga (ES)	FJ914402
	PT-FarP2I3-1-17	*Ipomoea indica*	Faro (PT)	MG254544
	PT-FarP2I3-2-17	*Ipomoea indica*	Faro (PT)	MG254545
SPLCD2	VE-1764E13-09	*Merremia dissecta*	Sucre (VE)	KF716173
SPLCD3	PR-T1_1-10	*Bemisia tabaci*/tomato	Santa Isabel (PR)	KT099179
ToLCD	AU-96	Tomato	Northern Territory (AU)	U74627
ToYLDD1	CU-404N1-10	*Sidastrum micranthum*	Matanzas (CU)	JN819495
ToYLDD2	CU-603N1-11	*Sidastrum micranthum*	Matanzas (CU)	KU232893

CrYVD, Croton yellow vein deltasatellite; DesLDD, Desmodium leaf distortion deltasatellite; MalCuV, Malvastrum leaf curl deltasatellite; SiGYVD1, Sida golden yellow vein deltasatellite 1; SiGYVD2, Sida golden yellow vein deltasatellite 2; SiGYVD3, Sida golden yellow vein deltasatellite 3; SPLCD1, sweet potato leaf curl deltasatellite 1; SPLCD2, sweet potato leaf curl deltasatellite 2; SPLCD3, sweet potato leaf curl deltasatellite 3; ToLCD, tomato leaf curl deltasatellite; ToYLCD1, tomato yellow leaf distortion deltasatellite 1; ToYLCD2, tomato yellow leaf distortion deltasatellite 2.

Table S4. List of primers used for real-time PCR to amplify the deltasatellite and viral DNA or plant reference genes.

Deltasatellite/virus/plant reference genes	Primer code	Primer sequence (5'-3')	Reference
SPLCD1	MA2713	GCTGATAAAGCTGTCGGAACTA	This work
	MA2714	GTAGCGGTTTGGGCATTTAAC	
SPLCV	MA2711	ACTTTCCTCTTGGCCTTCTTC	This work
	MA2712	GGCAACAGCAAACGCTATATTC	
ToLCNDV DNA-A	MA2809	GATAAAGACGCCACTCTCTTCTC	This work
	MA2810	GTGCGGTTGTTCCTACTACAT	
ToLCNDV DNA-B	MA2811	CTTGTGGAAGTCACGGAAGT	This work
	MA2812	GCGTGTATTGTTTGGAGATTGG	
SiGYVV DNA-A	MA2727	TCTGATGTCACTCGTGGTAATG	This work
	MA2728	GTTCGTGTGGTTCTTCAGTTTG	
SiGYVV DNA-B	MA2729	GACCTGTTGCCTGTACTCTATG	This work
	MA2730	TCAGCTCTGCCATGACTTAAC	
ToLDeV	MA2725	GATAGAGGGAGGAGTTGAGGAA	This work
	MA2726	CAGGAGAGGGACAGAGCTATAA	
BCTV	MA2751	CCAGTTATCGTCAGCTCTATCC	This work
	MA2752	GAGATACGGGCCAATCTATCAA	
PP2A (*N. benthamiana*)	MA2707	GACCCTGATGTTGATGTTCGCT	[38]
	MA2708	GAGGGATTTGAAGAGAGATTTC	
EF-1α (zucchini)	MA2824	GCTTGGGTGCTCGACAAACT	[39]
	MA2825	TCCACAGAGCAATGTCAATGG	

SPLCD1, sweet potato leaf curl deltasatellite 1; SPLCV, sweet potato leaf curl virus; ToLCNDV, tomato leaf curl New Delhi virus; SiGYVV, Sida golden yellow vein virus; ToLDeV, tomato leaf deformation virus; BCTV, beet curly top virus; PP2A, protein phosphatase 2A; EF-1α, elongation factor-1α.

Table S5. Estimate of the coefficient of evolutionary differentiation (d) for the sweet potato leaf curl virus (SPLCV) and sweet potato leaf curl deltasatellite 1 (SPLCD1) genomes obtained in this work and isolates previously reported from Spain. Standard error estimates (S.E.) were obtained by a bootstrap procedure (500 replicates). Analyses were conducted using the maximum composite likelihood model with the MEGA 7 program [31].

**Populations**	**SPLCV**		**SPLCD1**	
	**d**	**S.E.**	**d**	**S.E.**
Murcia – Granada	–	–	0.437	0.036
Murcia – Málaga	–	–	0.404	0.027
Murcia – Cádiz	–	–	0.569	0.069
Murcia – Canary Islands	–	–	0.551	0.048
Granada – Málaga	0.141	0.015	0.145	0.026
Granada - Cádiz	0.420	0.023	0.312	0.023
Granada – Canary Islands	0.196	0.019	0.254	0.041
Málaga – Cádiz	0.339	0.016	0.392	0.020
Málaga – Canary Islands	0.424	0.013	0.288	0.028
Cádiz – Canary Islands	0.685	0.025	0.471	0.043
All populations	0.453	0.011	0.486	0.021

–, not calculable due to availability of only one sequence from Murcia population.

Table S6. Data of virus quantification by real-time PCR ($2^{-\Delta\Delta Ct}$) used to generate Figure 7 with Graphpad Prism 6.0 software. Outlier values identified by the ROUT method are highlighted in black.

ToLCNDV_Nb_Exp1_DNA-A_-δsat	ToLCNDV_Nb_Exp1_DNA-B_-δsat	ToLCNDV_Nb_Exp2_DNA-A_-δsat	ToLCNDV_Nb_Exp2_DNA-B_-δsat	ToLCNDV_Nb_Exp1_DNA-A_+δsat	ToLCNDV_Nb_Exp1_DNA-B_+δsat	ToLCNDV_Nb_Exp2_DNA-A_+δsat	ToLCNDV_Nb_Exp2_DNA-B_+δsat
55,878.2803	1,732.9328	14,899.7448	1,491.9671	70,630.3194	3,099.8872	6,742.1827	609.7187
34,041.3351	942.2722	16,024.5919	1,024.7100	61,402.0839	1,584.7066	17,743.4631	1,079.3896
48,442.9801	2,567.2356	8,135.4137	883.4471	113,238.1061	4,045.2131	77,720.0926	7,326.9650
24,339.2991	781.4447	9,541.4975	707.2125	46,180.6225	1,628.1298	14,745.6316	1,396.8874
25,197.6226	483.0404	45,418.7380	927.3692	70,045.2694	2,253.5754	97,830.7241	3,856.2958
88,906.4212	**6,480.9968**	30,936.0204	1,622.4969	95,684.6579	12,704.0423	62,000.8339	2,460.9506
32,541.6549	1,391.0900	101,985.1803	4,585.9344	183,065.4486	10,311.7388	140,967.2007	6,281.9642
57,929.0747	3,514.2473	24,542.5919	422.2636	113,002.8787	7,141.4435	119,694.7210	5,556.6513
30,278.3717	1,032.5529	40,454.1475	1,288.0742	97,695.1959	4,793.9528	110,523.9881	5,412.2021
31,695.7108	1,216.0611	70,385.9571	3,350.1268	30,978.9366	1,158.4660	73,476.7401	3,125.7789
52,244.8061	2,948.9793	32,339.2814	843.9420	166,365.8736	12,442.5969	125,645.5456	7,945.9280
23,526.5149	1,063.7917	39,266.1900	1,865.0518	236,421.2192	21,769.1884	132,075.1937	7,231.1029
96,216.7201	**7,744.7341**	**179,173.7793**	5,828.8679	268,209.3489	24,971.5889	79,023.8218	2,795.7119
39,157.4718	2,367.2560	132,901.6969	4,426.6512	179,671.2438	6,912.5390	104,489.6123	3,251.7486
		49,667.0005	2,724.9235	151,294.4982	8,433.9924	141,751.0602	7,945.9280

ToLCNDV_zuc_Exp1_DNA-A_-δsat	ToLCNDV_zuc_Exp1_DNA-B_-δsat	ToLCNDV_zuc_Exp2_DNA-A_-δsat	ToLCNDV_zuc_Exp2_DNA-B_-δsat	ToLCNDV_zuc_Exp1_DNA-A_+δsat	ToLCNDV_zuc_Exp1_DNA-B_+δsat	ToLCNDV_zuc_Exp2_DNA-A_+δsat	ToLCNDV_zuc_Exp2_DNA-B_+δsat
11,730.6879	3,797.9363	23,987.5800	13,261.9013	8,192.0000	3,198.1015	16,013.4884	6,562.3644
**59,064.3503**	**43,538.3768**	20,199.0525	9,089.5931	7,383.0438	4,399.1223	27,193.9858	13,484.3653
8,773.8845	2,208.7279	12,899.2539	6,268.9148	9,108.5141	4,064.8883	27,250.5929	11,028.8902
7,766.2369	2,111.4231	13,663.1262	4,757.5398	3,396.8929	1,128.3508	25,673.6358	24,833.5002
3,769.0886	491.8246	17,718.8825	6,994.4747	2,828.8501	1,195.1699	13,873.0752	7,825.6778
**36,814.8448**	**32,227.3960**	24,696.1752	31,021.9123			18,331.0271	9,802.9483
7,956.9510	1,775.4881	17,499.1836	19,714.9168			38,325.0486	12,607.5471
2,554.8095	1,012.0045	14,282.8870	21,071.4212			24,936.9949	8,317.8791
**22,978.5477**	**17,008.8738**	23,526.5149	30,679.7690			45,734.6503	16,612.7121
8,516.2347	3,036.0924	20,867.9318	22,149.7071				
10,506.5398	4,783.9944	23,445.1193	4,252.2185				
8,983.1145	2,844.5801	29,799.4896	12,677.6524				
4,887.9032	1,805.2714	26,395.4200	10,148.6485				
6,626.3560	2,866.3519	28,172.5005	11,175.0991				
5,326.6028	2,286.6185	23,090.3113	11,331.0976				

SiGYVV_Nb_Exp1_DNA-A_-δsat	SiGYVV_Nb_Exp1_DNA-B_-δsat	SiGYVV_Nb_Exp2_DNA-A_-δsat	SiGYVV_Nb_Exp2_DNA-B_-δsat	SiGYVV_Nb_Exp1_DNA-A_+δsat	SiGYVV_Nb_Exp1_DNA-B_+δsat	SiGYVV_Nb_Exp2_DNA-A_+δsat	SiGYVV_Nb_Exp2_DNA-B_+δsat
503.9022	37.3750	522.0340	67.6492	205.3584	31.5594	68.9271	8.0556
506.0023	133.1584	306.7671	47.3076	147.7485	42.6065	216.3165	28.4430
429.9425	29.0809	531.1590	17.1246	152.8529	30.6327	111.1219	15.9115
834.6341	260.2943	701.8408	27.1711	132.7898	19.3868	113.8508	17.2677
926.7266	150.2270	543.8255	30.7817	84.2731	13.0864	154.2365	67.5086
713.1195	195.3612	595.1048	75.5835	43.1115	10.6295	129.2482	34.1071
627.7298	180.7686	562.6134	173.6454	141.8281	38.0810	98.8384	30.1273
870.6807	264.2940	406.1869	102.0406	125.0187	44.2008	192.4047	23.1510
1,181.1693	371.9591	496.9648	81.2333	261.9232	**109.0616**	266.8712	75.5312
580.4387	109.2887	471.7897	79.3413	86.1632	13.2507	115.5201	11.6641
250.2108	57.2816	301.0794	33.6142	**469.5061**	37.1941	122.2762	19.6438
515.2040	87.6086	559.1145	86.1632	128.0000	27.2088	97.0059	30.5903
891.4438	96.8044	774.4348	50.5626	**417.8960**	**159.2335**	105.4197	44.8800
874.9156	242.6949	724.0773	19.6847	143.3104	61.9486	106.6692	11.6318
1,328.8845	130.9616	585.2868	63.9113	195.4966	26.1910	34.4158	2.7876

ToLDeV_Nb_Exp1_-δsat	ToLDeV_Nb_Exp2_-δsat	ToLDeV_Nb_Exp1_+δsat	ToLDeV_Nb_Exp2_+δsat
4,311.5772	5,315.5380	429.3469	1,079.3896
4,630.6532	7,918.4371	1,780.4177	1,684.3777
3,620.5672	8,792.1483	1,054.2490	1,893.7104
5,134.4713	5,812.7293	1,144.1020	2,219.4708
5,028.8054	9,694.8308	3,310.8823	3,263.0378
4,617.8321	4,844.0566	1,718.5785	1,177.0827
5,685.2182	9,351.6162	3,254.0033	2,633.9276
8,112.8888	5,720.7953	1,897.6524	1,918.8151
10,155.6855	6,700.2537	2,734.3837	1,936.1835
6,221.2984	4,124.4899	2,136.4502	1,893.7104
5,673.4084	7,527.7343	2,872.3185	1,528.6051
5,988.5873	6,463.0525	1,225.3685	2,019.8046
6,378.4935	4,405.2250	2,239.5606	838.6936
8,451.5486	7,296.5562	4,332.5479	1,713.8201
5,138.0314	5,415.9549	2,813.2070	1,036.8561

BCTV_Nb_Exp1_-δsat	BCTV_Nb_Exp2_-δsat	BCTV_Nb_Exp1_+δsat	BCTV_Nb_Exp2_+δsat
5,804.6767	3,010.9437	9,708.2800	718.5775
7,885.5737	1,985.1055	15,403.8141	3,808.4810
6,418.4090	2,519.6367	6,080.6085	1,244.1977
17,817.4098	3,082.7453	6,152.6832	10,326.0438
6,308.1445	2,256.7017	8,169.3184	12,296.8400
4,898.0779	2,031.0359	3,104.1875	1,844.4820
15,286.8125	2,070.8395	9,390.5894	3,717.1985
5,400.9594	2,042.3296		2,734.3837
8,118.5142	7,616.9621		
**35,119.8728**	6,922.1285		
5,091.9410	**19,362.8004**		
26,158.6415	10,884.5942		
9,449.3541	5,198.9335		
16,543.7653	4,983.6948		
12,926.1050	7,517.3059		

SPLCV_Exp1_-δsat	SPLCV_Exp1_+δsat
1,743.7772	708.6847
2,986.0032	839.8571
4,121.6320	469.5061
1,677.3872	234.1031
896.4007	764.3025
946.1991	627.7298
784.7014	614.3853
3,541.1445	514.4903
4,414.3950	374.8059
1,023.2905	427.8615
1,854.7384	907.6549
937.7113	375.0658

Table S7. Summary of statistical analyses performed to evaluate the effect of sweet potato leaf curl deltasatellite 1 on accumulation of different helper geminivirids.

Virus	Plant host	Experiment	Genome component	δsat	Passed normality test	Statistical test	*p*-value
ToLCNDV	*N. b.*	Exp. 1	DNA-A	-	Yes	Welch's t test	0.0006 ***
				+	Yes		
			DNA-B	-	Yes	Welch's t test	0.0037 **
				+	Yes		
		Exp. 2	DNA-A	-	Yes	Welch's t test	0.0088 **
				+	Yes		
			DNA-B	-	No	Mann-Whitney test	0.0126 *
				+	Yes		
ToLCNDV	Zucchini	Exp. 1	DNA-A	-	Yes	Welch's t test	0.4883 n.s.
				+	Yes		
			DNA-B	-	No	Mann-Whitney test	0.9328 n.s.
				+	Yes		
		Exp. 2	DNA-A	-	Yes	Welch's t test	0.2023 n.s.
				+	Yes		
			DNA-B	-	Yes	Welch's t test	0.5094 n.s.
				+	Yes		
SiGYVV	*N. b.*	Exp. 1	DNA-A	-	Yes	Welch's t test	<0.0001 ****
				+	Yes		
			DNA-B	-	Yes	Welch's t test	0.002 ***
				+	Yes		
		Exp. 2	DNA-A	-	Yes	Mann-Whitney test	<0.0001 ****
				+	No		
			DNA-B	-	Yes	Welch's t test	0.0061 **
				+	Yes		
ToLDEV	*N. b.*	Exp. 1		-	Yes	Welch's t test	<0001 ****
				+	Yes		
		Exp. 2		-	Yes	Welch's t test	<0001 ****
				+	Yes		
BCTV	*N. b.*	Exp. 1		-	Yes	Welch's t test	0.3193 n.s.
				+	Yes		
		Exp. 2		-	No	Mann-Whitney test	0.6642 n.s.
				+	No		
SPLCV	*N. b.*	Exp. 1		-	Yes	Welch's t test	0.0025 **
				+	Yes		

δsat, deltasatellite; –, absence of the deltasatellite; +, presence of the deltasatellte. TolCNDV, tomato leaf curl New Delhi virus; SiGYVV, Sida golden yellow vein virus; ToLDeV, tomato leaf deformation virus; BCTV, beet curly top virus; SPLCV, sweet potato leaf curl virus. *N. b.*, *Nicotiana benthamiana*.
